# A versatile and robust cell purification system with an RNA-only circuit composed of microRNA-responsive ON and OFF switches

**DOI:** 10.1126/sciadv.abj1793

**Published:** 2022-01-05

**Authors:** Yoshihiko Fujita, Moe Hirosawa, Karin Hayashi, Takeshi Hatani, Yoshinori Yoshida, Takuya Yamamoto, Hirohide Saito

**Affiliations:** 1 Department of Life Science Frontiers, Center for iPS Cell Research and Application, Kyoto University, 53 Kawahara-cho, Shogoin, Sakyo-ku, Kyoto 606-8507, Japan.; 2 Department of Cell Growth and Differentiation, Center for iPS Cell Research and Application, Kyoto University, 53 Kawahara-cho, Shogoin, Sakyo-ku, Kyoto 606-8507, Japan.; 3 Institute for the Advanced Study of Human Biology (WPI-ASHBi), Kyoto University, Yoshida-Konoe-cho, Sakyo-ku, Kyoto 606-8501 Japan.; 4 Medical-risk Avoidance based on iPS Cells Team, RIKEN Center for Advanced Intelligence Project (AIP), Kyoto 606-8507, Japan.

## Abstract

Human induced pluripotent stem cells (iPSCs) are promising cell resources for cell therapy and drug discovery. However, iPSC-derived differentiated cells are often heterogenous and need purification using a flow cytometer, which has high cost and time consumption for large-scale purification. MicroRNAs (miRNAs) can be used as cell selection markers, because their activity differs between cell types. Here, we show miRNA-responsive ON and OFF switch mRNAs for robust cell purification. The ON switch contains a miRNA-target sequence after the polyadenylate tail, triggering translational activation by sensing the target miRNA. By designing RNA-only circuits with miRNA-ON and -OFF switch mRNAs that encode a lethal ribonuclease, Barnase, and its inhibitor, Barstar, we efficiently purified specific cell types, including human iPSCs and differentiated cardiomyocytes, without flow cytometry. Synthetic mRNA circuits composed of ON and OFF switches provide a safe, versatile, and time-saving method to purify various cell types for biological and clinical applications.

## Teaser

MicroRNA-responsive ON and OFF switches provide a safe and time-saving method for purifying a wide range of cell types.

## INTRODUCTION

Human embryonic stem cells (ESCs) and induced pluripotent stem cells (iPSCs) have self-renewal capacity and differentiate into various cell types, making them promising cell resources for drug discovery and regenerative medicine. Clinical studies using iPSCs have produced cardiac muscle cells and nerve cells transplanted into humans (*1*–*3*). However, it is necessary to purify the cells before transplantation, because residual iPSCs or other contaminating cells may form tumors or impair the normal functions of the transplanted cells.

Efforts have been made to select the target cells by introducing a gene marker, such as a fluorescent protein, downstream of the cell type–specific promoter using a plasmid or virus vector. Because DNA-based plasmids or viral vectors may be inserted into the genome or remain after transplantation, cells with these vectors are to be avoided in clinical practice. Small molecule– and culture condition–based cell isolation have also been reported (*4*, *5*) but are limited to the isolation of specific cell types. As a more practical approach, target cells can be selected and purified using a flow cytometer and fluorescence-activated cell sorting (FACS). However, the throughput of a flow cytometer is low, and it takes much time and cost to obtain enough cells for human transplantation. To resolve this problem, magnetic-activated cell sorting has been developed, which uses magnetic beads to rapidly generate a large number of cells (*1*, *6*–*8*). In these methods, however, because it is necessary to use an antibody that recognizes cell surface antigens for the purification, it is difficult to separate cells that do not express the antigen. Moreover, even if the surface antigen is present, it takes several months to establish a new antibody. Furthermore, because the cells need to be taken out from the culture dish and processed by a specific device, the contamination risk increases. Thus, there is an urgent need to develop a new cell sorting method that enables the purification of many cell types in a safe, versatile, and scalable manner.

**Fig. 1.**
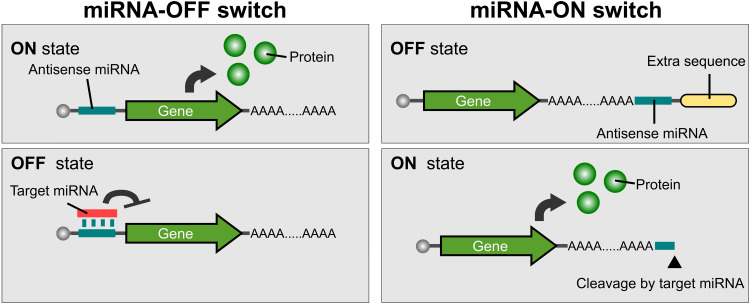
Design of endogenous miRNA-responsive switches. Schematic illustration of miRNA-OFF and -ON switches. The OFF switch (left) is translated in the same manner as normal mRNA (ON state) but inhibited (OFF state) in the presence of target miRNA. The ON switch (right) is composed of normal mRNA and an extra sequence downstream of the poly(A) tail connected by the antisense sequence of the target miRNA. In the absence of target miRNA, translation is suppressed because of the hidden poly(A) tail (OFF state). In the presence of target miRNA, the extra sequences are removed by miRNA-mediated mRNA cleavage to enhance translation (ON state).

The RNA switch is synthetic mRNA-based technology that controls the expression of any gene encoded on the exogenous mRNA by sensing intracellular biomolecules such as proteins or RNAs (*9*–*11*). MicroRNA (miRNA), which is a small-noncoding RNA that posttranscriptionally regulates the gene expression of target mRNAs, is a promising cell type selection marker because the activity and expression level of a miRNA are different between cell types (*12*). We have previously designed miRNA-responsive “OFF” switch mRNA (miRNA-OFF switch; Fig. 1, left) by repressing the expression of fluorescent reporter proteins or apoptotic proteins in response to endogenous miRNA levels, thus purifying specific cell types differentiated from human ESCs or iPSCs (*13*). In addition, the miRNA-OFF switch is mRNA-transcribed in vitro, so it should degrade rapidly in a cell, suggesting that it does not notably affect the level of endogenous miRNA, gene expression pattern, or cell viability (*13*). Moreover, using synthetic mRNA, the risk of insertion into the genome is low, suggesting that the RNA switch should be safe and eliminated at the time of the transplantation. Last, various miRNA-responsive switches can be easily prepared in a customized manner by changing the miRNA-targeting sequences and coding genes, making it possible to apply the technology to a broad range of cell purifications, classifications, and genome editing including human iPSCs and their differentiated cells (*13*–*16*).

However, to develop a robust and versatile cell purification system, we need to overcome several issues. First, the miRNA-OFF switch alone is not enough for the efficient purification of target cells because the efficiency of the translational repression is dependent on the activity of the endogenous miRNA in the target cells. For example, if the miRNA-mediated translational repression is weak, then some target cells to be enriched will be eliminated by the leaky expression of the apoptotic gene (e.g., *Bim*) encoded into the switch. Second, the number of miRNA-OFF switches introduced into the cells varies, so that the gene expression from each switch in each cell shows a broad distribution. Thus, because the balance of the number of endogenous miRNA molecules and OFF switches is important for efficient cell purification, optimization of the conditions for each experiment is necessary. In addition, the sensitivity to proapoptotic proteins such as Bim varies between cell types, which affects the efficiency of the cell purification using an apoptotic gene–encoding switch.

With the above points in mind, we aimed to develop miRNA-responsive “ON switch” mRNAs (miRNA-ON switches), which activate the translation of their own mRNA by sensing the target miRNA. Dual usage of miRNA-ON and -OFF switches should change the ratio of the protein production from each mRNA depending on only the endogenous miRNA level regardless of the amount of each mRNA switch in the cell. Use of the miRNA-ON switch also expands the ability of cell identification and separation, enabling the use of various miRNAs expressed in cells. In addition, the dual use of a cell death inducer and protector, which are independent of endogenous apoptotic pathways, in the ON and OFF switches, should invert a dominantly expressed gene between the target and nontarget cells in a robust manner. This is because the use of dual switches should block leaky gene expression irrespective of the different sensitivities to the apoptosis between cell types.

In this study, we first designed miRNA-ON switch that triggers translation activation by sensing the target miRNA expressed in various cell types. When we inserted the miRNA-target sequence and extra sequences downstream of the polyadenylate [poly(A)] tail, mRNA translation was inhibited. In the presence of the target miRNA, however, the translationally inhibitory sequence after the poly(A) tail was removed through miRNA-mediated target cleavage, generating translationally active mRNA in a cell type–specific manner. Thus, the mRNA functions as a miRNA-ON switch. To generate a robust cell purification system without a cell sorter, we then designed a synthetic mRNA-based circuit that is composed of both miRNA-ON and -OFF switches that encode the lethal ribonuclease (RNase), Barnase (Bn), and its inhibitory gene, Barstar (Bs). We purified cells of interest strictly using Bs as the inhibitor of leaky Bn expression in the target cell. In addition, Bn has the property of simultaneously degrading synthetic mRNA molecules introduced into the cells, making it possible to control the translation of cotransfected exogenous mRNA encoding for a drug-resistant gene in association with the Bn activity. That is, nontarget cells are eliminated strictly by not only the cytotoxicity of Bn itself but also that of the drug, thus enhancing the robustness and versatility of the cell purification system.

## RESULTS

### Design of miRNA-ON switch

We constructed a miRNA-ON switch that up-regulates translation in response to a target miRNA. It is known that a naturally occurring mRNA terminates at the poly(A) tail, and the translation is enhanced when the poly(A) tail is recognized by poly(A)-binding proteins. We introduced an antisense sequence of the target miRNA (anti-miRNA) downstream of the poly(A) tail and added an extra sequence (Ex) to inhibit the translational enhancement of the tail (Fig. 1, right; OFF state). Given that miRNA cleaves target mRNA with a perfectly matched sequence by slicer activity (*17*), we expected that the presence of the target miRNA should change the mRNA from an inactive to active form by cleaving it at the part of the miRNA recognition site to expose the translationally active poly(A) tail at the end (Fig. 1, right, ON state).

**Fig. 2.**
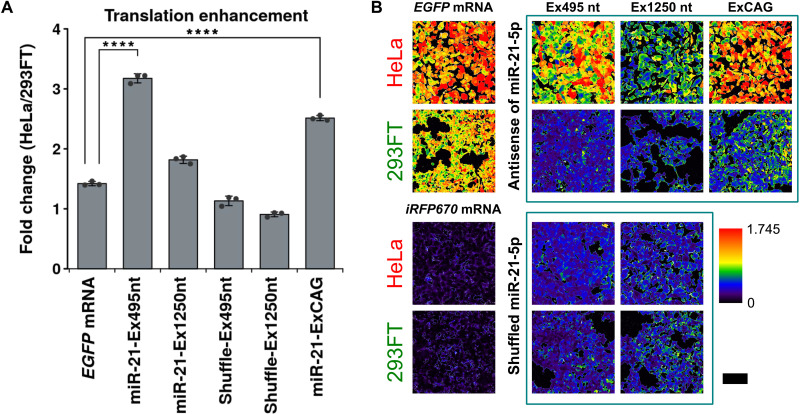
Translation efficiency of miR-21-5p–ON switches in HeLa and 293FT cells. (**A**) Fold change of the translation efficiency of the miR-21-5p–ON switch. “miR-21” indicates an ON switch having an antisense sequence for miR-21-5p, and “shuffle” indicates an ON switch having a shuffled antisense sequence for miR-21-5p. All values are shown as the ratios of the translation efficiency (EGFP/iRFP670) in HeLa to 293FT cells transfected with mRNA having extra sequences [495 nucleotides (nt), 1250 nt, or 30 CAG repeats] downstream of the poly(A) tail. Error bars represent means ± SD (*n* = 3), and data of each biological replicate are shown as a point. ****P < 0.0005 (Welch’s *t* test). (**B**) Ratiometric images of cells cotransfected with *iRFP670* mRNA and miR-21-5p–ON switch, which has the miR-21-5p antisense sequence or shuffled sequence. *iRFP670* mRNA was used as an internal control. The EGFP intensity at each pixel in a cell was divided by the iRFP670 intensity. Red ratiometric pseudo-color indicates a higher ratio (translation efficiency) of EGFP to iRFP670. Scale bar, 200 μm.

We chose miR-21-5p as the target miRNA, because miR-21-5p in HeLa cells shows strong activity and suppresses 90% of the miR-21-5p–OFF switch expression compared with 293FT cells (fig. S1) (*18*). We introduced an antisense sequence of miR-21-5p downstream of the poly(A) tail of *EGFP*-coding mRNA, followed by an additional sequence (see Materials and Methods; miR-21-Ex495nt: switch-*EGFP* mRNA) (Fig. 2A and table S1). *iRFP670*-coding mRNA as an internal control was cotransfected with switch-*EGFP* mRNA into HeLa cells and 293FT cells. Then, the ratio of enhanced green fluorescent protein (EGFP) to near-infrared fluorescent protein (iRFP)670 was observed by a flow cytometer and a fluorescence microscope. Compared with the control mRNA having the shuffled sequence of anti–miR-21-5p, EGFP expression from switch-*EGFP* mRNA (miR-21-Ex495nt) increased in HeLa cells but not in 293FT cells (Fig. 2, A and B, and fig. S2). We also designed mRNA with a longer sequence after the poly(A) tail (miR-21-Ex1250nt). This mRNA showed weaker translational activation compared with miR-21-Ex495nt (Fig. 2A). In addition, we designed mRNA with the CAG repeat (ExCAG) in 5′ untranslated region (5′UTR) and downstream of anti–miR-21-5p after poly(A) (table S1) (*19*). The protein expression from this mRNA also increased in HeLa cells compared with 293FT cells, and the expression level in HeLa cells (ON state) was comparable with the expression from the *EGFP* mRNA (Fig. 2A and B). On the basis of the obtained data, we used the miRNA-ON switch with ExCAG for the following studies.

**Fig. 3.**
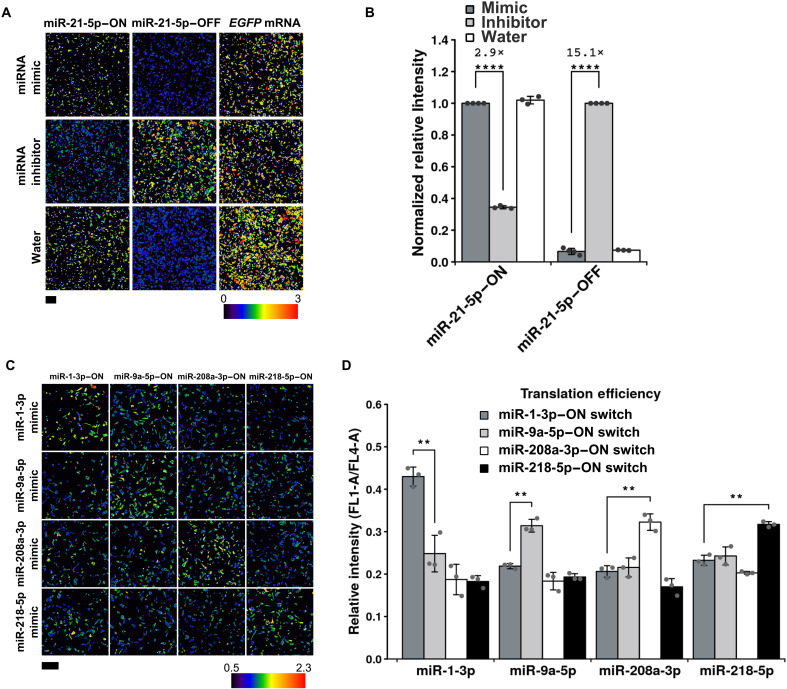
Response of miRNA-ON switches to various mimics and inhibitors. (**A**) Ratiometric images of HeLa cells cotransfected with miR-21-5p–ON and -OFF switches and a mimic/inhibitor of miR-21-5p. The EGFP intensity at each pixel in a cell was calculated using the same method as Fig. 2B. miR-21-5p–ON and –OFF indicate miR-21-5p–ON and -OFF switches, respectively. “*EGFP* mRNA” indicates mRNA encoding for EGFP without the anti–miR-21-5p sequence. (**B**) Normalized relative intensity (EGFP/iRFP670) of the miR-21-5p–ON and -OFF switches with miR-21-5p mimic or inhibitor. The relative intensities of ON and OFF switches were normalized by the value without either water and inhibitor, respectively. Error bars represent the means ± SD (*n* ≥ 3). Outliers were removed by Grubbs test. Data of each biological replicate are shown as a point. ****P < 0.0005. (**C**) Ratiometric images of HeLa cells cotransfected with various miRNA mimics and their ON switches. The EGFP intensity at each pixel in a cell was calculated using the same method as in Fig. 2B. The columns indicate each miRNA-ON switch, and rows indicate miRNA mimics. (**D**) Relative intensity (EGFP/iRFP670) of various miRNA-ON switches with mimics. Error bars represent the means ± SD (*n* = 3). Data of each biological replicate are shown as a point. **P < 0.01.

Next, we investigated whether the designed miRNA-ON switch could selectively activate translation by responding to target miRNA. We cotransfected miRNA switches with miRNA inhibitors or mimics into HeLa cells and confirmed the EGFP/iRFP670 ratio by fluorescence microscopy and a flow cytometer 1 day after the transfection (Fig. 3). As a control, we also transfected the miR-21-5p–OFF switch (Fig. 1, left), in which anti–miR-21-5p was inserted into the 5′UTR of the mRNA, to repress translation in the presence of miR-21-5p (Fig. 3A, middle column). EGFP/iRFP670 expression from the miR-21-5p–OFF switch was reduced to 10.1% in HeLa cells compared to 293FT cells (fig. S1) and 6.6% in miR-21-5p mimic–transfected cells compared to inhibitor transfected cells (Fig. 3B), confirming translational repression from the OFF switch. In contrast, higher EGFP/iRFP670 expression was observed with the miR-21-5p–ON switch under these conditions (Fig. 3A, left column). In addition, when the miR-21-5p inhibitor was added, the protein expression level was increased in cells transfected with the miR-21-5p–OFF switch, but it was decreased in cells transfected with miR-21-5p–ON switch (Fig. 3A, second row), confirming the opposing effect between ON and OFF switches that respond to endogenous miR-21-5p in HeLa cells (Fig. 3B). The fold change of the OFF switch (15.1-fold) between the miR-21-5p inhibitor– and mimic–transfected cells was higher than that of the ON switch (2.9-fold) (Fig. 3B). The addition of miR-21-5p mimic did not change EGFP/iRFP670 expression compared with the control water sample, indicating that the endogenous miR-21-5p level is high enough to control the switches in HeLa cells.

To investigate the versatility and selectivity of the miRNA-ON switch, we designed ON switches that respond to one of four different miRNAs (miR-1-3p, miR-9a-5p, miR-208a-3p, and miR-218-5p) by the same strategy and transfected the switches and miRNA mimics into HeLa cells. We observed that the EGFP signal was increased only when the mimic of the corresponding miRNA was transfected (Fig. 3C and D), suggesting selective translational activation by sensing the target miRNA. From these results, we concluded that we could design miRNA-ON switches in a customized manner, in which translation is triggered by sensing the target miRNA.

**Fig. 4.**
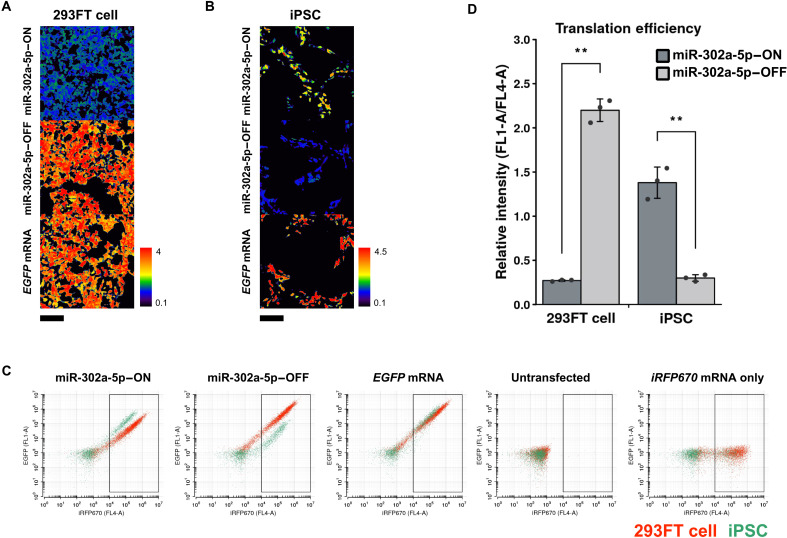
Response of miR-302a-5p–ON switches to endogenous miR-302a-5p in iPSCs. (**A** and **B**) Ratiometric images of 293FT and human iPSCs cotransfected with miR-302a-5p–ON and -OFF switches. The EGFP intensity at each pixel in a cell was calculated by the same method as in Fig. 2B. The translation efficiency (EGFP/iRFP670) of miR-302a-5p–ON switch was repressed in 293FT cells. In contrast, the translation efficiency was enhanced in iPSCs, which have high miR-302a-5p activity. In the case of OFF switch–transfected cells, the translation was repressed in iPSCs but not in 293FT. Scale bars, 200 μm. (**C**) Representative flow cytometry dot plots. 293FT and iPSCs cotransfected with miR-302a-5p–ON or –OFF switch are shown in red and green, respectively. Each cell population can be separated on the 2D plot. (**D**) Relative intensity (EGFP/iRFP670) of 293FT and iPSCs cotransfected with miR-302a-5p–responsive switches. Error bars represent the means ± SD (*n* = 3). Data of each biological replicate are shown as a point. **P < 0.01.

We next investigated whether the miRNA-ON switch could detect human iPSCs because it is necessary to identify iPSCs from other cell types for clinical cell transplantation. We constructed the miR-302a-5p–ON switch, which contains anti–miR-302a-5p after the poly(A) tail of the *EGFP*-encoded mRNA. The activity of miR-302a-5p is high enough to reduce the expression from the miR-302a-5p–OFF switch, which contains anti–miR-302a-5p at the 5′UTR of the mRNA, to 13.7% in iPSCs compared to 293FT cells (*20*, *21*). After transfecting the miR-302a-5p–ON or –OFF switch into human iPSCs (201B7 line) and 293FT cells, we confirmed that the miR-302a-5p–OFF switch repressed EGFP expression in the iPSCs, but not in 293FT cells and the miR-302a-5p–ON switch activated EGFP expression only in iPSCs (Fig. 4, A and B). A two-dimensional (2D) plot of the flow cytometry data showed iPSCs and 293FT cells as separate populations, confirming that the miR-302a-5p–ON switch clearly distinguished iPSCs (Fig. 4C). It is noteworthy that the miR-302a-5p–ON and –OFF switches exhibited opposite behavior against the target, miR-302a-5p, in iPSCs, indicating that the ON switch selectively activates translation by sensing miR-302a-5p, making it possible to distinguish iPSCs from other cells in which miR-302-5p is inactive through the fluorescent protein expression (Fig. 4D).

### Controlling Bn and Bs activity by miRNA-ON and OFF switches

We previously designed proapoptotic Bim protein-controllable miRNA-OFF switches to purify human iPSC-derived differentiated cells and showed that the switch is sufficient to purify some target cells (*13*). However, to control cell fate with a single miRNA-OFF switch that encodes a lethal gene, the amount of the switch must be adjusted tightly so that the expression level of the lethal gene is below the threshold for undesired target cell death (fig. S3). Current miRNA-OFF switches alone cannot completely suppress leaky expression of the lethal gene upon sensing the target miRNA because the level of translation from the switches is determined by the balance between them and the miRNA expressed in the cell. Because of the leaky expression of Bim in the cells to be enriched, efficient cell purification has remained a challenge for some cell types. In another example, cell classifier circuits encoding Bax selectively killed HeLa cells by sensing miR-21-5p; however, we found that nontarget 293 cells also died because of the leaky expression of Bax (*22*). In addition, previous studies demonstrated that miR-21-5p–responsive circuits can kill HeLa cells more easily than other cell types (e.g., 293FT cells). On the other hand, the survival of HeLa cells by eliminating other cell types has not been demonstrated (fig. S4).

To construct a more robust and efficient cell purification system, we designed an mRNA-based circuit that compensates for the OFF switch. The circuit contains both miRNA-ON and -OFF switches that encode Bn and its inhibitory protein, Bs. Bn is a lethal RNase that induces cell death by degrading RNAs, and Bs efficiently inhibits the RNase activity of Bn through a monomeric (Bs:Bn = 1:1) interaction (*23*, *24*).

The gene expression level of a transgene shows a wide distribution due to differences in the amount of transgenes (e.g., transfected mRNA) in each cell. Thus, if the lethal gene is controlled by a single OFF switch [e.g., Bim-OFF switch (*13*)], then the group showing a higher expression level of the lethal gene in the target cell population to be purified frequently overlaps with the nontarget cell population to be killed [green and red distributions between “overlap” in fig. S3 (top)], allowing some nontarget cells to survive. This phenomenon causes contamination and decreases the efficiency of the cell purification. If the amount of the transgene is adjusted so as to increase the purity, then a considerable number of target cells in the pink region [green distribution or dots within the overlap region in fig. S3 (bottom) or S5 (left)] will be eliminated because of leaky expression of the lethal gene.

In contrast, when two different mRNAs encoding *Bn* and *Bs* are introduced into the cell, the ratio of the *Bn*/*Bs* mRNA in each cell is expected to be roughly constant. We confirmed that the change of the dose dependency of the expression ratio of EGFP and iRFP670 from cotransfected cells was within 1.3-fold, but the EGFP expression itself increased about 2-fold (fig. S6A). Thus, the ratio of the Bn and Bs protein expression levels is kept constant and shows a belt-like distribution (figs. S5, right, and S6B) (*18*). The amount of active Bn is considered to be the expression level of Bn minus the expression level of Bs. When the expression level of Bn at which cells start to die is assigned as T, then cells die in the region satisfying Bn − Bs > T (fig. S5, right pink area).

Assuming that the ratio of Bn to Bs is constant in a cell, the gene expression levels of individual cells are distributed in the direction of the diagonal arrows (fig. S5, right). In this case, the cells remain in the same region, dead or alive, irrespective of the amount of mRNA transfected into the cells. This point is particularly important for the practical use of miRNA switches to purify cells because it is difficult to tightly control the amount of mRNA introduced into individual cells for various experimental conditions. Moreover, the use of miRNA-ON switch improves the cell purification, because miRNA expressed in target cells can directly activate translation of the cell death inhibitor, for instance. In this sense, the dual use of the miRNA-ON and -OFF switches should purify the target cells from a heterogeneous population in a more robust manner than the use of a single switch.

**Fig. 5.**
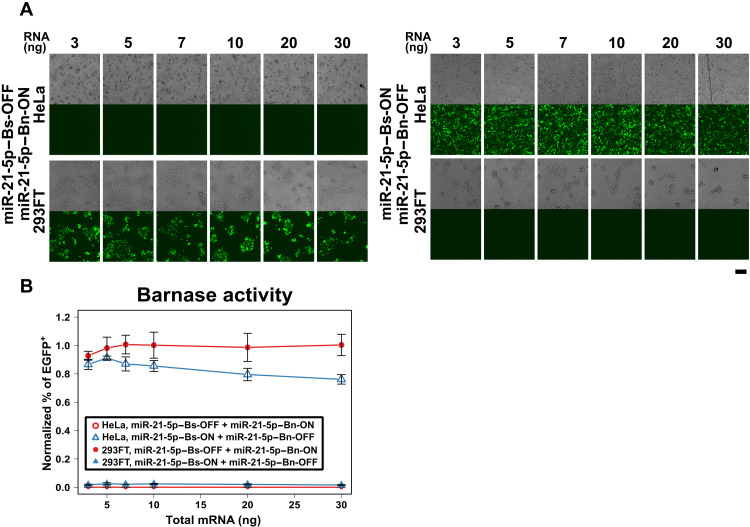
Robustness of miRNA-ON and -OFF switches encoding *Bn* and *Bs*. (**A**) Microscopic images of the cells transfected with miRNA-ON and -OFF switches. miR-21-5p–Bn-ON and miR-21-5p–Bs-ON indicate an miR-21-5p–ON switch encoding *Bn* and *Bs*, respectively. miR-21-5p–Bn-OFF and miR-21-5p–Bs-OFF indicate miR-21-5p–OFF switches encoding *Bn* and *Bs*, respectively. High Bn activity in HeLa cells cotransfected with miR-21-5p–Bn-ON and miR-21-5p–Bs-OFF degrades *EGFP* mRNA, resulting in no *EGFP* signal. In the case of 293FT, the Bn activity is repressed, causing EGFP-positive cells (left). In contrast, miR-21-5p–Bs-ON and miR-21-5p–Bn-OFF switches digest *EGFP* mRNA only in 293FT cells (right). Scale bar, 200 μm. (**B**) Dependency of Bn activity on the amount of transfected RNA. The values show the relative percentage of EGFP-positive cells cotransfected with RNA switches and *EGFP* mRNA normalized by the percentage of EGFP-positive cells transfected with *EGFP* mRNA only. Open circles indicate HeLa cells transfected with miR-21-5p–Bn-ON and miR-21-5p–Bs-OFF switches; open triangles indicate HeLa cells transfected with miR-21-5p–Bn-OFF and miR-21-5p–Bs-ON switches; and closed circles and triangles are 293FT cells cotransfected with the same switches as HeLa cells, respectively. The proportion of EGFP-positive cells is not drastically affected by the total amount of transfected RNA. Error bars represent the means ± SD of biological replicate (*n* = 3).

To confirm our hypothesis, we prepared synthetic mRNAs encoding *Bn* and *Bs* in miR-21-5p–ON and -OFF switches, respectively (miR-21-5p–Bn-ON and miR-21-5p–Bs-OFF), and transfected the switches into 293FT and HeLa cells. In the case of 293FT cells (low miR-21-5p activity), miR-21-5p–Bs-OFF and miR-21-5p–Bn-ON switches should induce Bs (ON) but inhibit Bn (OFF) expression. Even if a leaky expression of Bn is observed, Bs expression, which should be higher than Bn expression, blocks the Bn activity. In contrast, in the case of HeLa cells (high miR-21-5p activity), the Bs and Bn expressions should be OFF and ON, respectively. Bn expression, which is higher than the leaked Bs expression, should induce efficient cell killing (fig. S7). To monitor the activity of Bn in cells, we cotransfected *EGFP* mRNA with these switches. If the activity of Bn is high in a cell, then the transfected *EGFP* mRNA should be degraded rapidly, repressing EGFP expression (Fig. 5A and fig. S7).

To observe the robustness of the system, we cotransfected various amounts of miR-21-5p–ON and –OFF switches (3 to 30 ng) with *EGFP* mRNA into HeLa and 293FT cells and quantified the EGFP fluorescence to analyze intracellular Bn activity. First, to determine the appropriate ratio of Bn to Bs, we introduced miR-21-5p–Bn and -Bs switches into the cells (total of 7 ng per well; 96-well plate in total) at various ratios and observed the EGFP expression and cytotoxicity. Then, we selected ratios showing different EGFP expressions between HeLa and 293FT cells (fig. S8, red rectangles). When miR-21-5p–Bn-ON and miR-21-5p–Bs-OFF switches (HeLa killing) were transfected into the cells, EGFP fluorescence was only observed in 293FT cells. In contrast, when miR-21-5p–Bn-OFF and miR-21-5p–Bs-ON switches (HeLa-survival) were transfected, EGFP fluorescence was only observed in HeLa cells (Fig. 5, A and B). Notably, we observed that the fluorescence intensity was maintained irrespective of the amount of transfected RNA, confirming that the effect of dual ON and OFF switch selection (fig. S5) is robust against the amount of total mRNA switch in each cell and cancels any leaky expression of Bn.

### Purification of specific cell types and elimination of undesired cells using dual ON/OFF switches

**Fig. 6.**
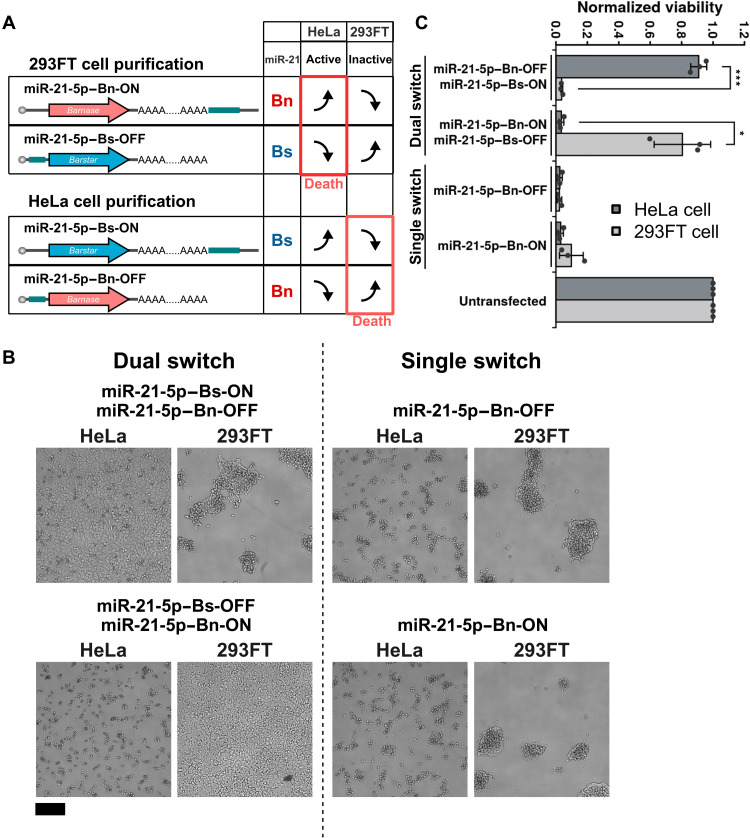
Induction of cell type–specific cell death by miRNA-ON and -OFF switches. (**A**) Schematic illustration of the combination of switches that efficiently purify miR-21-5p–active (HeLa) or –inactive (293FT) cells. The translation of miR-21-5p–ON switch encoding *Bn* and OFF switch encoding *Bs* increases and decreases, respectively, in HeLa cells due to high miR-21-5p activity, resulting in the activation of Bn and thus the promotion of cell death. In contrast, the translation of Bn and Bs decreases and increases in 293FT cells, respectively, resulting in the survival of 293FT cells. HeLa cells can also be purified using miR-21-5p–Bs-ON and miR-21-5p–Bn-OFF switches. (**B**) Microscopic images of each cell 2 days after transfection. miR-21-5p–Bn-ON, miR-21-5p–Bs-ON, miR-21-5p–Bn-OFF, and miR-21-5p–Bs-OFF are the same as in Fig. 5B. In the case of the dual switch using miR-21-5p–Bs-ON and miR-21-5p–Bn-OFF (top left), 293FT cells showed an abnormal shape and detached from the bottom of the well. In the case of miR-21-5p–Bs-OFF and miR-21-5p–Bn-ON (bottom left), HeLa cells detached from the well. Both cell types transfected with either single miR-21-5p–Bn-OFF or miR-21-5p–Bn-ON switch showed abnormal morphology. Scale bar, 200 μm. (**C**) Cell viability by the WST-1 assay was performed 2 days after the transfection. The absorbance values were subtracted by the absorbance of blank wells and normalized by the absorbance of untransfected cells. Error bars represent the means ± SD (*n* = 3), and data of each biological replicate are shown as a point. *P < 0.05 and ***P < 0.001.

Next, we investigated whether the combination of miRNA-ON and -OFF switches could purify cells of interest by eliminating undesired cell types. Using HeLa and 293FT cells, we tested whether either cell type could be removed or enriched using the miR-21-5p–ON and -OFF switches efficiently. When the pair miR-21-5p–Bn-ON and miR-21-5p–Bs-OFF is introduced into these cells, Bn activity should only appear in HeLa cells, inducing selective HeLa cell death (Fig. 6A, 293FT cell purification). On the other hand, when the pair miR-21-5p–Bs-ON and miR-21-5p–Bn-OFF switches is introduced, Bn activity should only appear in 293FT cells, inducing 293FT cell death (Fig. 6A, HeLa cell purification).

We transferred the switch pairs shown in Fig. 6A into HeLa and 293FT cells and observed the cells by microscopy. The cells with high Bn activity showed abnormal morphology (Fig. 6B), whereas the cells with negligible or no Bn activity showed normal morphology. As expected, the combination of both ON and OFF switches selectively and efficiently killed the target cells exclusively (either HeLa or 293FT cells). In contrast, transfection of either single miR-21-5p–Bn-OFF or miR-21-5p–Bn-ON switch promoted cell death in both cell types, likely due to the leaky expression of Bn. Cell viability assays confirmed the nontarget cell killing and target cell purification by the pairs, but not by either switch alone (Fig. 6, B and C). The transfection of the miRNA switches did not notably reduce the viability of the target cells (Fig. 6C), consistent with our previous report (*13*).

In addition, we investigated whether the transfection of mRNA and miRNA switches affects gene expression profiles including those of immune response–related genes, by RNA sequencing (RNA-seq). A principal components analysis divided each cell type into different groups regardless of the transfected RNA species, verifying that the RNA transfection had no effect on the gene expression profile specific to the cell type. However, HeLa cells showed more variation in gene expression than 293FT cells (fig. S9, A and B). In addition, a gene ontology (GO) analysis of HeLa cells showed that interferon- and antiviral-related genes were induced by the RNA transfection (fig. S9E). On the other hand, the difference in induction between *EGFP* mRNA and the Bn/Bs switch transfection was smaller than the difference between transfection and no transfection, suggesting that the choice of the RNA does not create the dominant effect (fig. S9, C and D). In addition, *EGFP* mRNA rapidly decreased over time (fig. S9F), falling below 10% in 3 days, indicating that the effect of the mRNA transfection is transient.

**Fig. 7.**
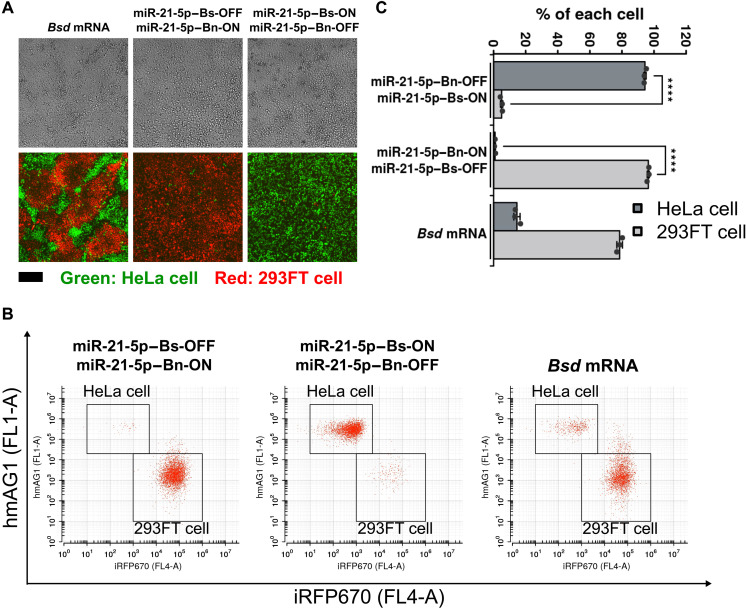
Purification from mixed cells by miRNA-ON and -OFF switches. (**A**) Microscopic images of cocultured HeLa and 293FT cells 3 days after the transfection. HeLa cells stably expressing hmAG1-M9 and 293FT cells stably expressing iRFP670-M9 were used to distinguish each cell line. Top images are bright-field images, and bottom images are merged images of hmAG1 and iRFP670. Green shows the hmAG1 signal, and red shows the iRFP670 signal. Scale bar, 200 μm. (**B**) Representative 2D flow cytometry plots. The squares indicate the gates of each cell. (**C**) Percentage of each cell. The number of each cell type was counted inside the squares in (B), and the percentage of each cell line was calculated. Error bars represent the means ± SD (*n* = 3), and data of each biological replicate is shown as a point. ****P < 0.0005.

Next, we mixed HeLa and 293FT cells and aimed to purify either cell type during coculturing in one dish. To distinguish the cells, we generated stable cell lines that express distinct fluorescent proteins [human azami green (hmAG) with a nuclear localization signal, M9, in HeLa cells (hmAG1-M9) and iRFP670 with M9 in 293FT cells (293FT-iRFP670-M9)] (*25*). When we performed cell purification with the corresponding ON and OFF switch pairs, only one expected fluorescence (either HeLa or 293FT) was observed (Fig. 7A). By measuring the ratio of HeLa and 293FT cells with a flow cytometer, we found that the target cells were enriched to more than 95% yield (Fig. 7, B and C). It is noteworthy that we could efficiently purify HeLa cells by eliminating 293FT cells, which are known to be resistant to apoptosis (*26*–*28*), due to the effects of Bn.

To compare the efficiency of combining ON and OFF switches encoding *Bn* and *Bs* in this study with a previously reported OFF switch (*13*), we analyzed the performance of the miR-21-5p–Bim-OFF switch in 293FT and HeLa cells. We expected Bim to be expressed in 293FT cells to induce cell death but repressed in HeLa cells due to miR-21-5p–mediated regulation. However, we found that 293FT cells were not killed efficiently, and HeLa cells were not purified no matter the amount of miR-21-5p–Bim-OFF switch (fig. S4). We assumed that because 293T cells are less sensitive to apoptosis than HeLa cells (*26*–*28*), it was difficult to purify HeLa cells even if the expression level of Bim in 293FT cells is higher than that in HeLa cells. In contrast, the combination of ON and OFF switches encoding *Bn* and *Bs* successfully enriched the target cells (Fig. 7), showing better cell purification performance than the Bim-OFF switch alone.

### Robust and efficient purification of human iPSCs and iPSC-derived cardiomyocytes without a cell sorter

**Fig. 8.**
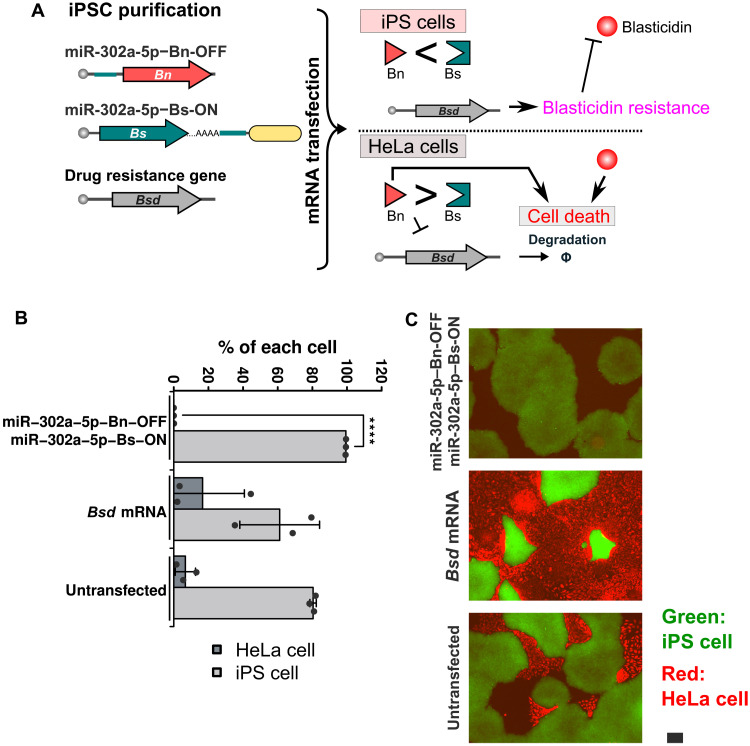
Purification of iPSCs from a cell mixture. (**A**) Schematic illustration of the double selection of iPSCs. miR-302a-5p–Bn-OFF and miR-302a-5p–Bs-ON indicate miR-302a-5p–OFF and -ON switches encoding Bn and Bs, respectively. The switches and mRNA encoding *Bsd* are cotransfected into heterogeneous cell populations that include the target cells. In iPSCs, Bn activity is repressed, gaining the cells drug resistance. In contrast, Bn activity increases in HeLa cells, resulting in the degradation of *Bsd* mRNA. Consequently, HeLa cells lose drug resistance and are removed by not only Bn activity but also blasticidin. (**B**) Percentage of iPSCs and HeLa cells after transfection followed by one passage. The number of cells for each cell type was counted inside the squares in fig. S11, and the percentage of each cell line was calculated. Error bars represent the means ± SD (*n* = 3), and data of each biological replicate is shown as a point. ****P < 0.0005. (**C**) Merged fluorescence images of the cells treated with switches followed by passaging once. *GFP*-integrated human iPSC line (iPS-EGFP) and HeLa-iRFP670-M9 are colored in green and red, respectively. Scale bar, 200 μm.

To investigate the versatility of the miRNA-sensing ON and OFF switches, we next aimed to purify human iPSCs by eliminating contaminated cells. We cotransfected mRNA encoding a blasticidin resistance gene, blasticidin-S deaminase (*Bsd*), with iPSC-selective ON and OFF switches and added blasticidin to the medium to select mRNA-transfected cells and eliminate mRNA-untransfected cells (Fig. 8A). Given that the expression of *Bsd* mRNA is suppressed by Bn activity in contaminated cells, not only the removal of untransfected cells but also the double selection of Bn and blasticidin to eliminate undesired cells can be performed. To distinguish between iPSCs and contaminated cells (e.g., HeLa cells), we used strains stably expressing EGFP (green: iPSCs) and iRFP670 (red: HeLa). We cocultured the cells, transfected them with miR-302a-5p–Bn-OFF and miR-302a-5p–Bs-ON switches, and then measured their percentages with a flow cytometer. We observed that iPSCs were enriched to approximately 95% using the miR-302a-5p–ON and –OFF switches, whereas untransfected samples contained a higher percentage (57%) of HeLa cells (fig. S10). We found that the purity of iPSCs could exceed 99%, by a single passage of cells, confirming the survival of the iPSCs and removal of residual HeLa cells (Fig. 8, B and C, and fig. S11). Thus, it is likely that the contaminating cell population (~5%) just after the selection by the switches were dead cells not released from the bottom of the well due to insufficient cell washing. In addition, we observed normal iPSC colonies, proliferation, and the normal expression of the pluripotency surface marker TRA-1-60 after the purification, indicating negligible side effects of the switches on iPSCs (Fig. 8C and fig. S12D). We also performed RNA-seq to investigate the gene expression profile of the iPSCs treated with the same purification process. We quantified the potential target genes of miR-302a-5p predicted by TargetScan (*29*) or miRTarBase (*30*) and the genes required for pluripotency (*31*, *32*). As a result, the expression of miR-302a-5p–targeted genes was maintained, but that of pluripotency-associated genes was slightly decreased after 72 hours, and the expression levels of both miR-302a-5p target and pluripotency-associated genes were similar to control iPSCs after 120 hours (fig. S12, A and B). The transgene of *Bsd* mRNA also decreased rapidly over time, similar to *EGFP* mRNA (figs. S9F and S12C).

**Fig. 9.**
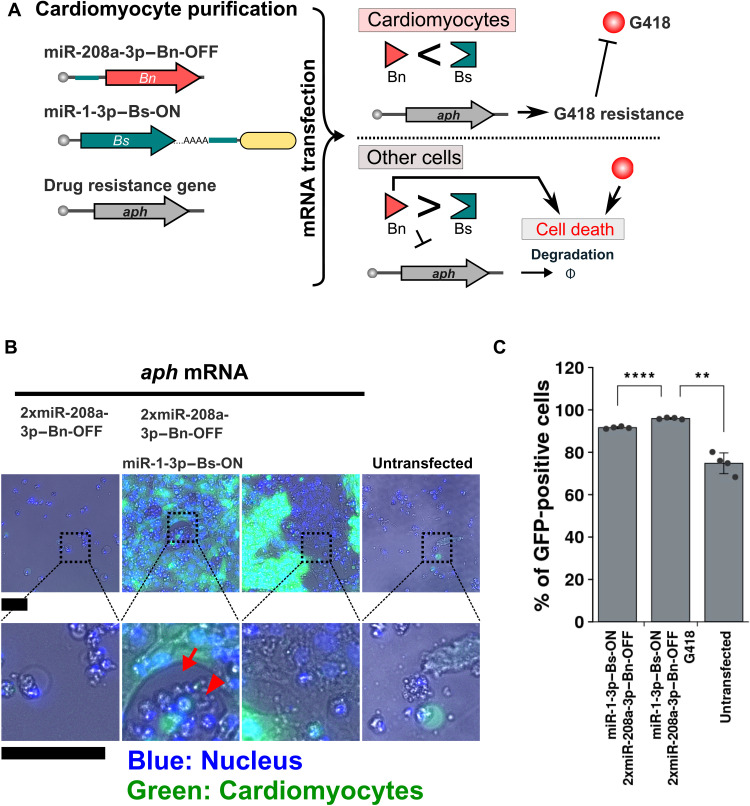
Purification of iPSC-derived cardiomyocytes. (**A**) Schematic illustration of the double selection of iPSC cardiomyocytes. miR-208a-3p–Bn-OFF and miR-1-3p–Bs-ON indicate miR-208a-3p–OFF and miR-1-3p–ON switches encoding *Bn* and *Bs*, respectively. The switches and mRNA encoding G418, the resistance gene (*aph*), are cotransfected to a heterogeneous cell population that includes the target cells. In the case of cardiomyocytes, Bn activity is repressed, gaining the cells drug resistance. In contrast, Bn activity increases in nontarget cells, resulting in the degradation of drug-resistant mRNA. Consequently, nontarget cells lose the drug resistance and are removed by not only Bn activity but also by G418. (**B**) Merged microscopic images of heterogeneous cells treated with the switches and G418. Nuclei stained with Hoechst are indicated in blue, and cardiomyocytes expressing GFP are indicated in green. Bottom row, merged images areas of the broken squares in the top images. The red arrowhead shows shrunken cells. The red arrow indicates an area with no cells due to the removal of nontarget cells. Scale bars, 200 μm. (**C**) Percentage of iPSC cardiomyocytes counted inside the gate in fig. S13. Error bars represent the means ± SD (*n* = 4), and data of each technical replicate are shown as a point. **P < 0.01 and ****P < 0.0005.

Last, we differentiated cardiomyocytes from human iPSCs (iPSC cardiomyocytes) and purified them using cardiomyocyte-active miRNA (miR-208a-3p or miR-1-3p)–sensing Bs-ON and Bn-OFF switches. It is important to purify iPSC cardiomyocytes without using a flow cytometer because large amounts of cardiomyocytes are required for transplantation and heart therapies. We previously showed that miR-1-3p– and miR-208a-3p–Bim-OFF switches purified iPSC cardiomyocytes, although the purification efficiency was approximately 90% (*11*). To confirm whether the combination of ON and OFF switches can enhance the cardiomyocyte purification, we designed miR-1-3p– and miR-208a-3p–sensing Bn-OFF and Bs-ON switches (Fig. 9A). A PEST sequence, which is a signal peptide of protein degradation (*33*), was added to Bn so that Bn is degraded faster than Bs. iPSC cardiomyocytes were identified using *GFP* downstream of the MYH6 promoter. After differentiating the iPSCs to cardiomyocytes for approximately 2 weeks, miR-208a-3p–Bn-OFF and miR-1-3p–Bs-ON switches were introduced into the cell populations. RNA encoding a G418-resistance gene, aminoglycoside-3′-phosphotransferase (*aph*), was also introduced into the cells. The mRNA-untransfected cells were removed by adding G418 to the medium, as were unwanted cells (not iPSC cardiomyocytes), in which the expression of aph was suppressed by Bn. The GFP-positive iPSC cardiomyocytes were measured with a flow cytometer or a fluorescence microscope (Fig. 9, B and C, and fig. S13). After the selection by the switches, we confirmed that a certain number of GFP-negative cells other than iPSC cardiomyocytes remained on the wells, but the cells detached from the bottom of the wells and their shapes were abnormally rounded (Fig. 9B, red arrow and arrowhead). Thus, we considered the cells dead, indicating that the iPSC cardiomyocyte purification efficiency was increased during the cell culture, similar to the iPSC purification (Fig. 8B and fig. S10B). The purity of the GFP-positive cells after washing was increased to 91.6% by introducing the ON and OFF switches. Last, we analyzed the purity of the cardiomyocytes by immunostaining for cardiac isoform of troponin T (cTNT), finding that cTNT-positive cells were 94.8% after treatment with the switches (fig. S14).

## DISCUSSION

In this study, we designed a new miRNA-ON switch by inserting an anti-miRNA sequence and extra sequence after the poly(A) tail of the mRNA (Figs. 1 to 4). Translational activation by the mRNA was triggered by sensing the target miRNA. We assumed that the extra sequence after the poly(A) tail inhibited the formation of translationally active mRNA in the cell such that the removal of the inhibitory sequence caused by miRNA-dependent RNA cleavage may recruit poly(A)-binding proteins more effectively, making it possible to form active mRNA ready for translation. However, the exact molecular mechanism of ON switches needs further investigation in the future. Ultimately, we created a robust and versatile cell purification system composed of synthetic mRNA-based ON and OFF switches that encode a lethal RNase, *Bn*, and its inhibitor, *Bs* (Fig. 5 and figs. S5 and S7).

With regard to translation, the OFF switch was more potent than the ON switch (Fig. 3B), but leakage could not be suppressed completely. To preserve this potency and strictly suppress undesired cell death by minimizing the leaky activity of Bn with Bs, we combined miR-21-5p–Bn-OFF and –Bs-ON switches, which enabled us to purify HeLa cells without markedly reducing the cell survival rate (Fig. 6, B and C). This result is in sharp contrast to using a single miR-21-5p–OFF switch encoding an apoptosis gene (*Bim*) (fig. S4), which failed to eliminate 293FT cells, thus lowering the purification efficiency of HeLa cells. It is also likely that 293FT cells are less sensitive to Bim-dependent apoptosis pathways and are more robust to cell death than HeLa cells. In addition, by pairing the ON and OFF switches, strict control of the amount of mRNA is not needed (Fig. 5, A and B). Thus, it is possible to stably control Bn activity at a wide range of transfected mRNA levels (one magnitude difference, 3 to 30 ng), which is desired for practical use, since the transfection efficiency of mRNA is dependent on various parameters such as the cell type, number of cells, and cell condition.

Our system can regulate translation from synthetic mRNAs by controlling RNase activity. That is, we can control mRNA activity by tuning the RNase and its inhibitor produced from the mRNA switches. Compared with other cell-killing genes such as apoptotic genes, Bn can regulate RNA-based circuits itself. The cotransfected exogenous mRNA, including drug resistance genes, can be regulated by the Bn activity. For future cell therapies, we applied our system to purify two different types of cells, human iPSCs and iPSC cardiomyocytes. We confirmed that both cell types were efficiently selected from heterogeneous populations without a cell sorter. It is noteworthy that the purity of the cells after selection was approximately 95% (Figs. 7C and 9C, and fig. S10B), but the purity after one passage was more than 99% in the case of iPSCs (Fig. 8B). We assumed that the residual 5% of the population was due to dying or dead cells that adhered to the wells (Fig. 9B) and not removed during the washing. Thus, the cell purification efficiency is improved by cell culturing.

Overall, our method can remove mRNA-untransfected cells and cells resistant to Bn simultaneously and is applicable to a wide range of cell types for high purity. However, the yield of the cells may depend on the cell type. While we found that proliferative cells such as HeLa and 293FT cells maintained comparable viability after selection (Fig. 6C), the yield of cardiomyocytes still needs improvement; we estimated that the cardiomyocyte yield after purification was 28% the starting number of cardiomyocytes (fig. S14C). To improve the yield, several issues need to be overcome, including the mRNA transfection efficiency, optimization of the ratio of the Bn and Bs switches, and the appropriate choice of a commercial drug and its concentration as the second selection agent, because the drug resistance gene-encoded mRNA introduced together with the switches could be degraded by Bn. Nevertheless, the results indicate that miRNA-ON and -OFF switches can be used for cell types beyond cardiomyocytes intended for use in transplantations. The next challenge is to increase the fold change of the current ON switch to the same level as the OFF switch by optimizing the structure and sequences introduced after the poly(A) tail of the mRNA. In addition, the RNA transfection efficiency in 3D cell cultures such as organoids should be improved to control cell fate in tissues.

The synthetic mRNA system shown in this study has a low risk of inserting foreign RNA into the genome. Although the RNA transfection of miRNA-Bn/Bs-ON and -OFF switches affected the gene expression profiles of the purified cells to some extent (figs. S9 and S12), the effect diminished over time. The transgene also decreased rapidly, down to less than 10% in 3 days, indicating that the perturbation caused by miRNA switches is transient and could be acceptable for future cell therapy. In addition, in contrast to identifying cells with antibodies and analyzing them one by one with a flow cytometer to separate them, cells can be processed at the same time so that expensive equipment, which has a contamination risk, is not required. This feature is suitable for scaling up. Thus, our mRNA-based cell purification system could provide a versatile technique in cell preparations for transplantation and regenerative medicine in the future.

## MATERIALS AND METHODS

### Plasmid construction

Synthesized 5′UTR and 3′UTR oligo DNAs were used for generating the pUC19 vector (TaKaRa) including UTRs prepared using the In-Fusion HD Cloning Kit (TaKaRa). Once the vector-containing UTRs was constructed, we prepared the linearized vector including 5′UTR and 3′UTR by inverse polymerase chain reaction (PCR) and inserted only the open reading frame (ORF) into the vector by the In-Fusion HD Cloning Kit to construct pUC19 vectors including the 5′UTR, ORF, and 3′UTR. Bs was amplified by fusion PCR of six synthetic oligo DNAs (YF472, YF473, YF474, YF475, YF476, and YF477) and digested by Nco I and Bgl II. The digested fragment was cloned into the pSM vector previously constructed (*18*) or a pCM vector, which has the same sequence as the pSM vector except for the drug resistance gene, and sequenced. Then, the ORF of Bs was amplified using the primers YF783 and YF804 and cloned into the same linearized vector described above. Bn was amplified from pFN19K HaloTag T7 SP6 Flexi Vector (Promega) using the primers YF770 and YF807. Because the leaky expression of Bn is lethal for *Escherichia coli*, we fused Bs amplified using the primers YF805 and YF806 to the Bn fragment amplified using the primers YF770 and YF807 to cancel the Bn activity. After the fusion PCR, the fragment including Bs and Bn was cloned into the linearized vector using the In-Fusion HD Cloning Kit. The sequences of synthesized oligo DNAs are listed in table S2, and the combinations of primers and DNA fragments for PCR are shown in table S3.

To construct piggyBac vectors for the establishment of cell lines stably expressing fluorescent proteins, KW148_PB53-CAG-*GFP*-puro (gift from K. Woltjen) was linearized by inverse PCR using the primers YF812 and YF813. The hmAG1-M9 and iRFP670-M9 fragments were amplified from pUC19-*hmAG1-M9* and pUC19-*iRFP670*-*M9* using the primers YF810 and YF811 and cloned into the linearized vector using the In-Fusion HD Cloning Kit. p*iRFP670*-N1 was a gift from V. Verkhusha (Addgene plasmid #45457; http://n2t.net/addgene:45457; RRID:Addgene_45457) (*34*).

### Cell culture

HeLa cells and HeLa cells stably expressing hmAG1 and a nuclear localization signal M9 (HeLa-hmAG1-M9) were cultured in Dulbecco’s modified Eagle’s medium (DMEM; Nacalai Tesque) supplemented with 10% fetal bovine serum (FBS). 293FT cells and 293FT cells with iRFP670 and M9 stably expressed (293FT-iRFP670-M9) were cultured in DMEM supplemented with 10% FBS (JBS), nonessential amino acids (Thermo Fisher Scientific), 1 mM sodium pyruvate (Sigma-Aldrich), and 1 mM l-glutamine (Thermo Fisher Scientific). HeLa cells stably expressing iRFP670 (HeLa-iRFP670) were cultured in DMEM supplemented with 10% FBS and puromycin (1 μg/ml). A wild-type human iPSC line (201B7), *GFP*-integrated human iPSC line (iPS-EGFP) (*35*), and MYH6-*GFP*–integrated human iPSC line (iPS-MYH6-GFP) (*36*) were cultured in StemFit AK02N (Ajinomoto) according to a feeder-free culture system (*37*). Mixtures of HeLa-hmAG1-M9 and 293FT-iRFP670-M9 cells were cultured in the same medium used for the 293FT cells. Mixtures of HeLa-iRFP670 and iPS-EGFP were cultured in StemFit AK02N medium. iPSC-derived cardiomyocytes were maintained and seeded in differentiation medium 4 (see “Cardiomyocyte differentiation” section for the medium constituents) according to the protocol previously described (*38*).

### Generation of stable cell lines

HeLa and 293FT cells were seeded in 12-well plates in appropriate medium the day before transfection. The seeded cells were cotransfected with PiggyBac vectors containing *hmAG1-M9* or *iRFP670-M9* and 200 ng of the PiggyBac transposase vector pCAG-PBase (gift from K. Woltjen) (*39*) using Lipofectamine 2000 (Thermo Fisher Scientific). At 1 day after the transfection, the medium for HeLa and 293FT was replaced with the appropriate medium described above supplemented with puromycin (0.7 and 1 μg/ml; Invivogen), respectively. The cells were cultured in the medium supplemented with puromycin for 1 to 2 weeks until untransfected cells were not detected by fluorescence microscopy or flow cytometry.

### Cardiomyocyte differentiation

To differentiate iPSCs into clumps of cardiomyocytes, we modified a previously reported protocol (*38*) to reduce the population of cardiomyocytes before purification. StemPro-34 supplemented with 2 mM l-glutamine, l-ascorbic acid (50 μg/ml), transferrin (150 μg/ml), and 0.4 μM monothioglycerol was used as the basal medium. Briefly, feeder-free iPS-MYH6-GFP cells were suspended in differentiation medium 1 [basal medium supplemented with 0.5% (v/v) Matrigel matrix, 10 μM Y-27632 (Wako), and recombinant human BMP4 (2 ng/ml)] in a 100-mm ultralow attachment culture dish or 96-well clear round-bottom ultralow attachment microplate (Corning). On day 1, an equal volume of differentiation medium 2 [basal medium supplemented with rhBMP4 (18 ng/ml), recombinant human Activin A (12 ng/ml), and recombinant human basic FGF (10 ng/ml)] was added to the dish. On day 3, the medium was replaced with differentiation medium 3 [basal medium supplemented with 0.5% (v/v) Matrigel matrix, 1 μM stemolecule Wnt Inhibitor https://www.reprocell.com/product-catalog/small-molecules/stemolecule-wnt-inhibitor-iwp-3, and recombinant human VEGF (10 ng/ml)]. On day 7, the medium was replaced with differentiation medium 4 [basal medium supplemented with rhVEGF (5 ng/ml)]. After day 10, the medium was replaced with fresh differentiation medium 4 every few days.

### mRNA preparation

Template DNAs for in vitro transcription were amplified by PCR (TOYOBO) using appropriate synthesized oligo DNAs (Eurofin or Fasmac; see tables S2 and S3). The template of the mRNA for simply expressing a gene was amplified from a vector containing the 5′UTR, ORF, and 3′UTR using primers with the T7 promoter (YF771) and poly(A) tail (KEC883). The template of the miRNA-OFF switch was amplified from the same plasmid using KEC879, KEC883, and a primer containing an anti-miRNA sequence in the 5′UTR. For the miRNA-ON switch, the ORF, poly(A) tail, miRNA antisense sequence, and extra sequence were fused in this order to prepare the template for in vitro transcription. The templates were purified using SpeedBeads magnetic carboxylate modified particles (GE Healthcare) or the MinElute PCR Purification Kit (QIAGEN). The RNAs were transcribed for about 4 hours at 37°C using the MEGAScript T7 Transcription Kit (Thermo Fisher Scientific) as previously described (*13*). To escape an immune response and enhance translation, we used 1-methylpseudouridine-5′-triphosphate, Ψ, instead of uridine-triphosphate, U, and Antireverse Cap Analog, ARCA (TriLink). In the iPSC purification from the iPSC and HeLa coculture experiments, we used pseudouridine-5′-triphosphate and 5-methylcytidine-5′-triphosphate (TriLink Bio Technologies) instead of natural UTP (uridine triphosphate) and CTP (cytidine triphosphate), respectively. The transcribed RNAs were purified using Monarch RNA Cleanup Columns (NEB), RNeasy MinElute Cleanup Kit (QIAGEN), or SpeedBeads magnetic carboxylate modified particles (GE Healthcare). The RNAs were treated with TURBO deoxyribonuclease (DNase; Thermo Fisher Scientific) and Antarctic Phosphatase (NEB) or rAPid Alkaline Phosphatase (Roche).

### mRNA transfection

HeLa, 293FT, iPSCs, and their modified cell lines expressing fluorescent proteins were seeded into a multi-well plate the day before transfection. Transcribed RNAs were transfected into the cells using Lipofectamine MessengerMAX (Thermo Fisher Scientific) according to the manufacturer’s protocol. Cell clumps after at least 13 days of cardiomyocyte differentiation were transferred to conical tubes and harvested after the cell clumps naturally fell down. The harvested cell clumps were resuspended in collagenase I solution (2 mg/ml) supplemented with DNase I (10 μg/ml) and rotated for more than 2 hours at 37°C. After the incubation, the supernatant was removed, and the pellet of cell clumps was resuspended in Accumax (Nacalai Tesque) and incubated for about 30 min at 37°C. Then, the cell suspension was pipetted to dissociate the clumps to single cells and diluted by fresh differentiation medium 4. The dissociated cell suspension was centrifuged for 5 min at room temperature, and the supernatant was aspirated. The cells were resuspended in differentiation medium 4 and counted using Countess II (Thermo Fisher Scientific). The transfection conditions are listed in table S4.

### Cell proliferation assay

On the day before transfection, 1 × 10^4^ of HeLa-hmAG1-M9 and 293FT-iRFP670-M9 cells were seeded onto 96-well plates. The RNA switches and mRNA-encoding *Bsd* were transfected with Lipofectamine MessengerMAX (0.2 μl per well). At 4 hours after the transfection, an equal volume of medium supplemented with blasticidin-S (60 μg/ml; Invivogen) was added to each well to remove untransfected cells. Fluorescence images of the cells were captured using the Cytell Cell Imaging System (GE Healthcare) after 2 days of culture. Medium supplemented with ^1^/_10_ of WST-1 reagent (Roche Diagnostics KK) was prepared and used to replace the medium of the transfected cells. After incubation at 37°C for 1 hour, absorbance was measured at 440 and 620 nm by a microplate reader (Infinit M1000, Tecan). The data were subtracted by the value of blanks and normalized by untreated samples by R.

### Observation of fluorescence images and image processing

Cell images were captured using an IX81 flurorescence microscope (Olympus), In Cell Analyzer 6000 (GE Healthcare), Cytell Cell Imaging System (GE Healthcare), or CQ1 confocal image cytometer (Yokogawa Electric Corporation). The image processing was performed using an ImageJ plugin (https://github.com/yfujita-skgcat/image_converter).

### Flow cytometry analysis

The medium for HeLa, 293FT, and their derived cells was washed by phosphate-buffered saline (PBS) once and replaced with 30 μl of Accumax (Nacali Tesque), followed by incubation at 37°C for about 10 min. After the confirmation of cell detachment from the bottom of the wells, the cells were resuspended in an additional 70 μl of DMEM supplemented with 10% FBS. The suspended cells were analyzed using an Accuri C6 flow cytometer (BD Biosciences) and FL1 filter (530/30 nm, 90% attenuation) for hmAG1, EGFP, and FL4 filter (675/25 nm) for iRFP670. The data were extracted from the Flow Cytometry Standard (FCS) files, which were generated by the equipment, using the “flowCore” package of R (*40*) and analyzed using the package “mgcv.” Outliers were removed using the package “outliers.” The mean number of cells analyzed in each experiment is shown in table S5.

### iPSC purification from iPSC and HeLa cell coculture

iPS-EGFP and HeLa-iRFP670 cells were seeded in laminin-coated 24-well plates in StemFit AK02N containing Y-27632 (Wako) (ratio, iPS:HeLa = 3:2; total, 1.2 × 10^5^ cells per well). Before transfection, the medium was changed to StemFit AK02N without Y-27632. miR-302a-5p–Bn-OFF switch, miR-302a-5p–Bs-ON switch, and blasticidin resistance mRNA (*Bsd* mRNA) were transfected using Lipofectamine MessengerMAX Transfection Reagent (Thermo Fisher Scientific) according to the manufacturer’s protocol. At 4 hours after the transfection, the culture medium was changed to StemFit AK02N containing blasticitidin (160 μg/ml). Before the flow cytometry analysis, the cells were washed with PBS and captured by the Cytell Cell Imaging System (GE Healthcare Life Sciences). At 3 days after the transfection, the cells were washed with PBS, treated with 200 μl of Accumax (Funakoshi), and incubated at 37°C with 5% CO_2_ for 10 min. The samples were analyzed on the Accuri C6 Flow Cytometer with FL1 filter (533/30 nm) and FL4 filter (675/25 nm). We defined iPSCs and HeLa cells as FL1-A (EGFP)– and FL4-A (iRFP670)–positive populations, respectively. For passages, after capturing the cell images, the cells were washed with PBS, treated with 200 μl of Accumax, and incubated at 37°C with 5% CO_2_ for 10 min. The cells were collected into 1.5-ml tubes, centrifuged at 200*g* for 5 min at 24°C, had the supernatant aspirated, mixed with StemFit containing Y-27632, and seeded in laminin-coated six-well plates. Seven days after the seeding, the cells were analyzed on the Accuri C6 Flow Cytometer as described above.

### Immunostaining for iPS-EGFP cells

For the immunostaining, iPS-EGFP cells were treated as described below. The cells were washed with PBS, treated with 200 μl (for 24-well plate) or 300 μl (for 6-well plate) of Accumax, and incubated at 37°C with 5% CO_2_ for 10 min. The cells were collected into 1.5-ml tubes, centrifuged at 200*g* for 5 min at 24°C, had the supernatant aspirated, mixed with StemFit containing Y-27632, and were counted. Cells (1.0 × 10^5^) were collected into 1.5-ml tubes [containing 500 μl of FACS buffer (PBS containing FBS, 30% glucose solution, and penicillin-streptomycin solution)] from the cell suspension and centrifuged at 200*g* for 5 min at 24°C. After the supernatant was discarded, 97.5 μl of FACS buffer and 2.5 μl of Alexa Fluor 647 mouse anti-human TRA-1-60 antigen (BD Biosciences) were added, and the mixture was incubated for 30 min on ice with protection from ambient light. After the incubation, 500 μl of FACS buffer was added, and the mixture was centrifuged at 200*g* for 5 min at 24°C. After removing the supernatant, 800 μl of FACS buffer was added, and the mixture was centrifuged again. After removing the supernatant and adding 200 μl of FACS buffer, the cells were analyzed on an Accuri C6 flow cytometer. We measured TRA-1-60 using the FL4 filter. In this experiment, only iPS-EGFP cells were seeded on laminin-coated 24-well plates (7.2 × 10^4^ cells per well). Seeding, transfection, and passages were the same as described in “iPSC purification from iPSC and HeLa cell coculture” section above.

### Purification of iPSC-derived cardiomyocytes

Embryoid bodies after more than 14 days of cardiomyocyte differentiation were collected and treated with collagenase [collagenase I (2 mg/ml; Sigma-Aldrich) and DNase I (10 μg/ml; EMD MilliPore)] at 37°C for 2 hours, followed by treatment with Accumax at 37°C for 30 min. The cells were dissociated by pipetting and washed once by differentiation medium 4. The RNAs listed in table S4 were reverse-transfected into the cells, which were then seeded onto a multiwell plate coated with fibronectin (Sigma-Aldrich). After 4 hours of transfection, an equal volume of medium supplemented with G418 (400 μg/ml) was added to remove untransfected cells and cells other than cardiomyocytes. The cells were incubated at 37°C with 5% CO_2_ for 3 days while changing to medium supplemented with G418 (200 μg/ml). The nuclei were stained using Hoechst 33342 (10 μg/ml; Thermo Fisher Scientific) for 30 min before capturing images using the Cytell Cell Imaging System. The cells were washed by PBS twice to remove dead cells and treated with AccuMax at 37°C for more than 10 min before flow cytometry analysis. For the cTNT assay, the cells were harvested and dissociated into single cells by Accumax. The cells were fixed by 4% paraformaldehyde for 20 min at room temperature, followed by two PBS washes. Then, the cells were permeabilized by 0.2% Triton X-100 for 20 min at room temperature. Troponin was detected by incubating with mouse anti–troponin T MS-295-PD as a primary antibody, followed by Alexa Fluor 546 goat anti-mouse (Life Technologies) as a secondary antibody at room temperature. The cells were analyzed on FACSAria II (BD Biosciences).

### RNA sequencing

HeLa cells were transfected with the miR-21-5p–Bn-OFF and miR-21-5p–Bs-ON switches, and 293FT cells were transfected with the miR-21-5p–Bs-OFF and miR-21-5p–Bn-ON switches at 1 day after seeding. The cells were harvested at 24, 48, and 72 hours after transfection and stored at −80°C until use. The total mRNA was adjusted to 100 ng by *EGFP* mRNA. iPSCs were transfected with the miR-302a-5p-Bn–OFF and miR-302a-5p-Bs–ON switches in addition to *Bsd* mRNA and selected by the same method as explained above for the iPSC purification from the HeLa cell coculture. The iPSCs were harvested at 72, 96, and 120 hours after the transfection and stored at −80°C until use. RNA was extracted using the mirVana miRNA Isolation Kit (Thermo Fisher Scientific) according to the manufacturer’s protocol. The RNA-seq libraries were prepared using the TruSeq Stranded mRNA Library Prep (Illumina) with IDT for Illumina TruSeq RNA UD Indexes (Illumina), quantified using the Qubit dsDNA HS Assay Kit (Thermo Fisher Scientific), and sequenced on a NextSeq500 (Illumina) as 76–base pair single reads.

### RNA-seq data analysis

The sequences obtained by NextSeq500 were trimmed and filtered using fastp 0.12.4 (*41*). The human genome, UCSC hg38 August 2015, was downloaded from the illumine Ready-To-Use Reference Sequences and Annotation web site (https://jp.support.illumina.com/sequencing/sequencing_software/igenome.html). The reads were mapped to the customized human genome, including *EGFP* and *Bsd* gene, by STAR 2.7.9a (*42*), and the uniquely mapped reads were counted using htseq-count 0.13.5 (*43*). The normalized read counts were calculated using DESeq2 R packages (*44*). For the GO analysis, the genes whose expression level was three times higher than in the untransfected sample with an adjusted *P* < 0.05 were selected using DESeq2 and analyzed using the clusterProfiler package (*45*). Pluripotency critical genes and the human ESC essentialome were obtained from (*31*) and (*32*), respectively. The candidate target genes of miR-302a-5p were predicted using TargetScan (*29*) and miRTarBase (*30*).

## Notes

View/request a protocol for this paper from *Bio-protocol*.
