# Supplementary material for: A versatile and robust cell purification system with an RNA-only circuit composed of microRNA-responsive ON and OFF switches
Source: Sci Adv. 2022 Jan 5;8(1):eabj1793. doi: 10.1126/sciadv.abj1793 (PMC8730616; doi:10.1126/sciadv.abj1793)
Supplement: Supplementary file 1 — Figs. S1 to S14 Tables S1 to S5 [file sciadv.abj1793_sm.pdf]

Science Advances
AAAS

Supplementary Materials for

A versatile and robust cell purification system with an RNA-only circuit composed of microRNA-responsive ON and OFF switches

Yoshihiko Fujita*, Moe Hirosawa, Karin Hayashi, Takeshi Hatani, Yoshinori Yoshida, Takuya Yamamoto, Hirohide Saito*

*Corresponding author. Email: hirohide.saito@cira.kyoto-u.ac.jp (H.S.); yoshihiko.fujita@cira.kyoto-u.ac.jp (Y.F.)

Published 5 January 2022, *Sci. Adv.* **8**, eabj1793 (2022)
DOI: 10.1126/sciadv.abj1793

This PDF file includes:

Figs. S1 to S14
Tables S1 to S5

Supplementary Figure 1

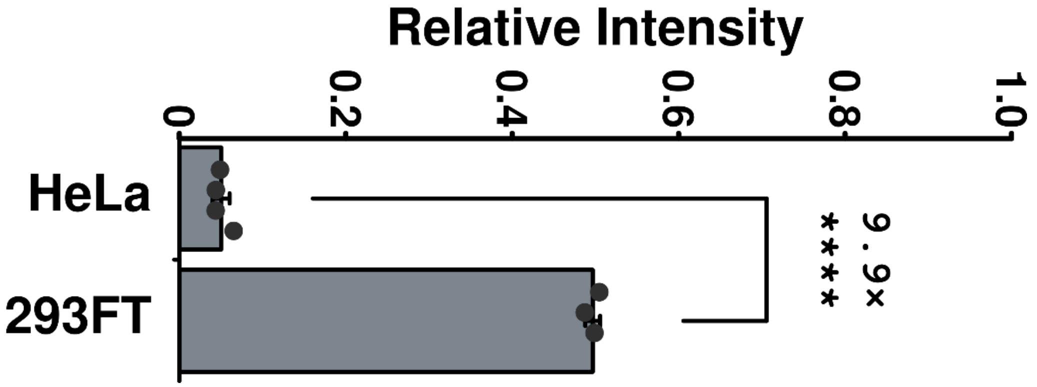

Fig. S1. Relative miRNA activity in HeLa cells and 293FT cells.

Relative intensity (EGFP/iRFP670) of miR-21-5p-OFF switch in HeLa cells and 293FT cells. Error bars represent the mean ± SD (n≥3). Outliers were removed by Grubbs test. The data of each biological replicate is shown as a point. ****P < 0.0005.

Supplementary Figure 2

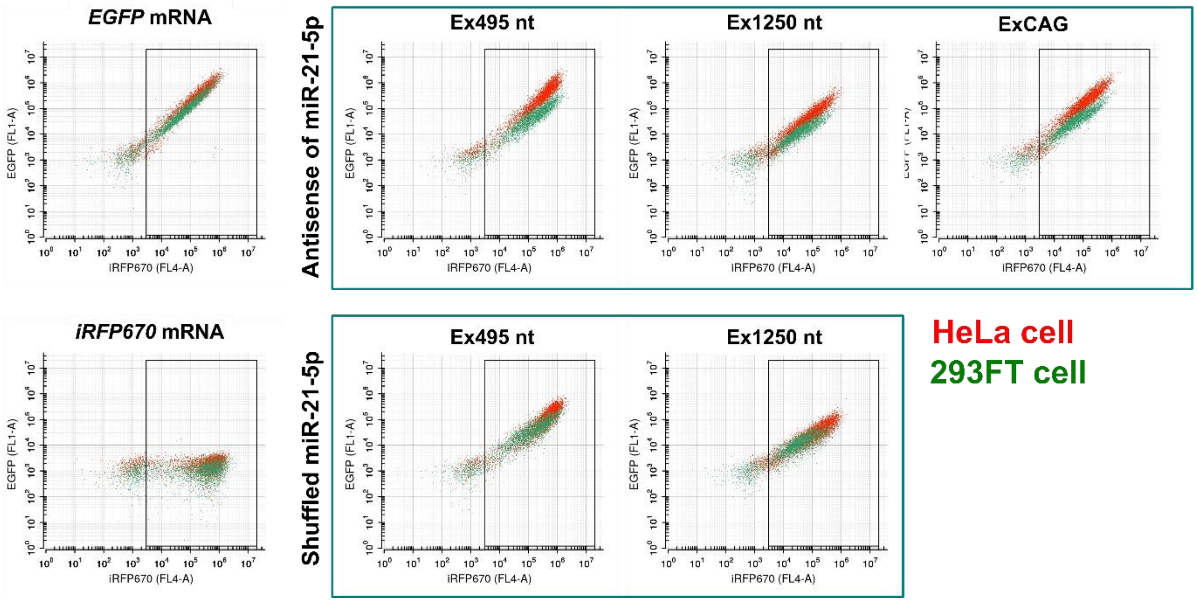

Fig. S2. Scatter plots of miR-21-5p-ON switch against endogenous miRNA

Cells co-transfected with *iRFP670* mRNA and miR-21-5p-ON switch that contains the miR-21-5p anti-sense sequence or shuffled sequence. *iRFP670* mRNA was used as the internal control. HeLa cells and 293FT cells are indicated as red and green dots, respectively.

Supplementary Figure 3

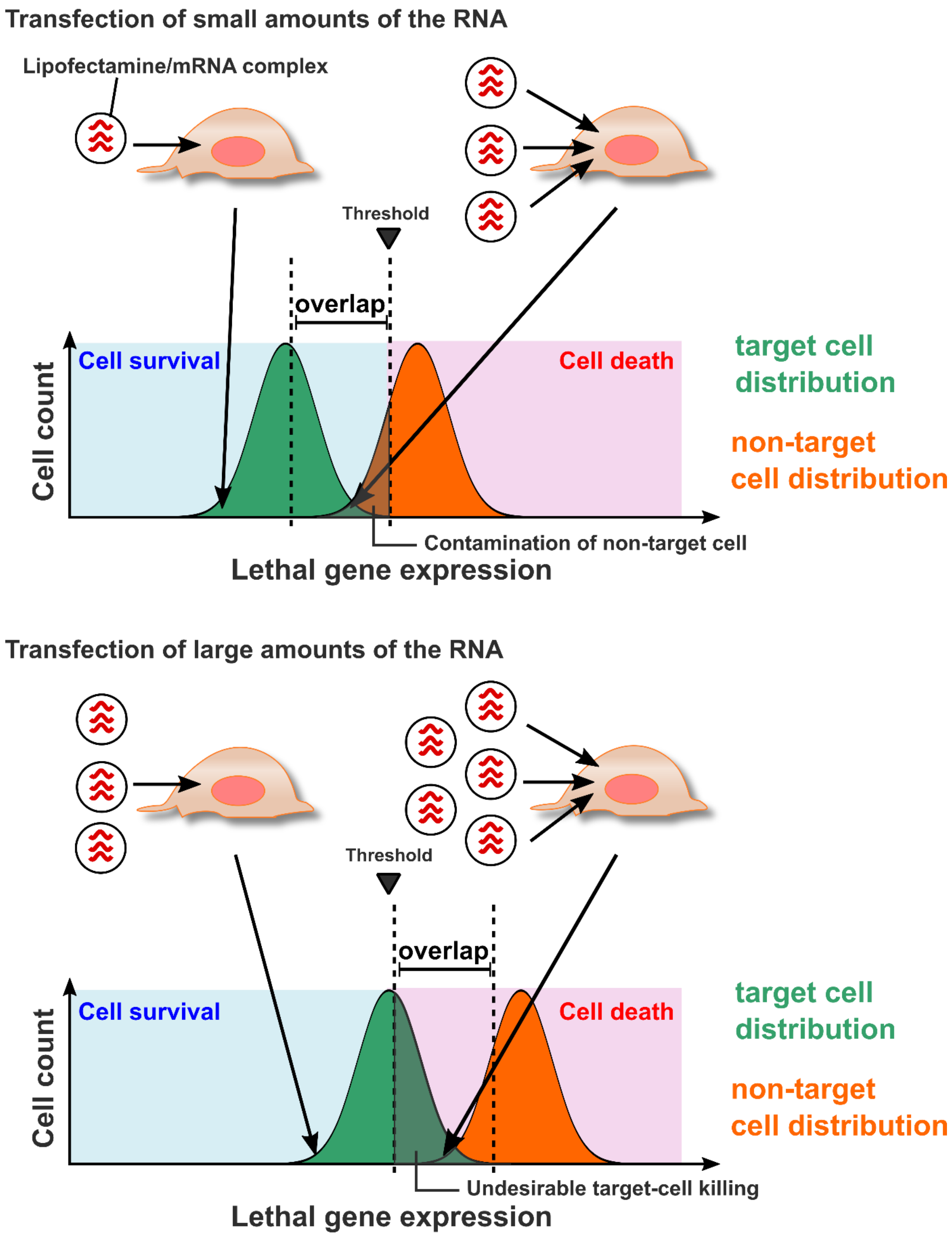

Fig. S3. Schematic illustration of the gene expression distribution in each cell type.

The expression level of the lethal gene from the switch in target cells (survive) is lower than that in non-target cells (death), because the endogenous miRNA expression shows a shifted distribution (green and orange distributions). The amount of introduced mRNA into individual cells is variable and difficult to be controlled precisely. In addition, the translation activity depends on the cell status, such as size, cell cycle, and health, resulting in a wide distribution of the expression level in each cell. Thus, the expression level in the target cells overlaps that in non-target cells (overlap). When a small amount of the switch is transfected to a heterogeneous cell population, the lethal gene is expressed in non-target cells, but not to the lethal threshold in some, resulting in contamination by non-target cells. To remove the contamination, we need to increase the amount of the switch transfected. Higher amounts of the transfected switch shift the expression distribution in both target- and non-target cells towards a higher lethal gene expression level (rightward). In this situation, the purity of the target cells increases but also results in a lower yield, because the expression level of the lethal gene in some target cells exceeds the lethal threshold.

Supplementary Figure 4

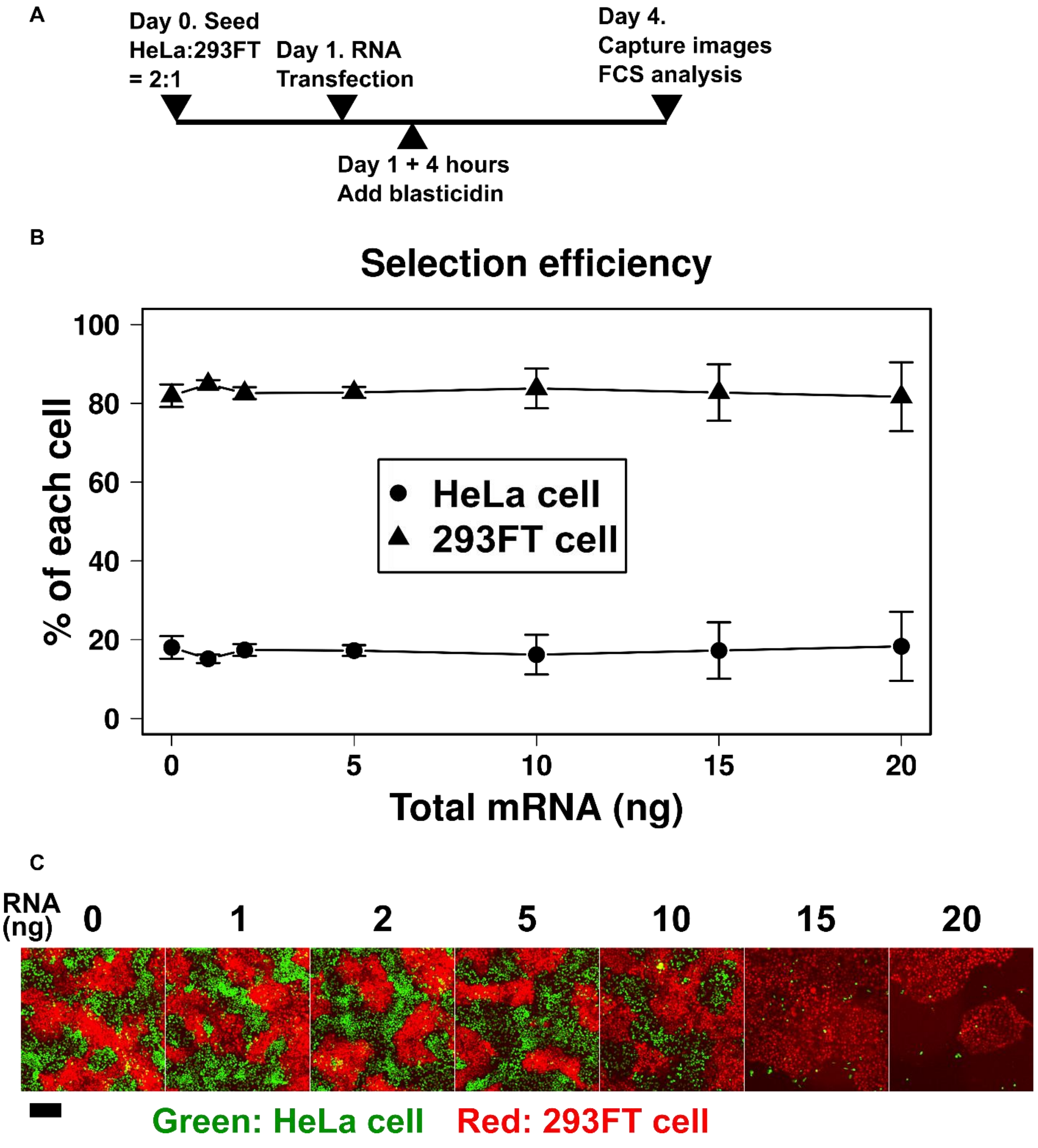

Fig. S4. The performance of the miR-21-5p-OFF switch encoding the pro-apoptotic gene ,*Bim*, to purify HeLa cells.

(A) Timeline of the purification of HeLa cells from a co-culture of HeLa and 293FT cells by miR-21-5p-Bim-OFF switch. **(B)** Dose-dependency of the selection efficiency of miR-21-5p-Bim-OFF switch. Co-cultured HeLa and 293FT were treated with miR-21-5p-OFF switch encoding the apoptotic protein Bim. The number of cells for each cell type was counted in a similar manner to Fig. 7B and 7F, and the percentage of each cell line was calculated. Closed

circles and triangles are HeLa and 293FT, respectively. Error bars represent the mean ± SD (n=3), and the data of each biological replicate is shown as a point. **(C)** Merged fluorescence microscopic images of co-cultured cells. HeLa cells with a stable and high expression of hmAG1-M9 and 293FT cells with a stable and high expression of iRFP670-M9 were used in order to distinguish the two cell lines. The co-cultured cells were transfected with miR-21-5p-OFF switch encoding *Bim*. HeLa cells were expected to survive, because high miR-21-5p activity should decrease the Bim expression. However, both cells were removed by the switch. Scale bar indicates 200 μm.

Supplementary Figure 5

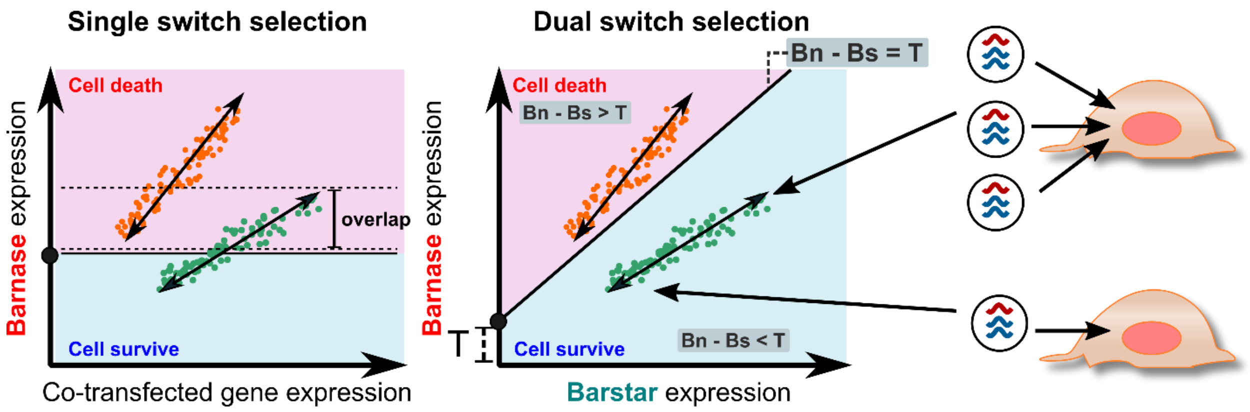

Fig. S5. Phase diagram showing the relationship between cell death and the expression levels of Barnase (Bn) and Barstar (Bs) using single- or dual-switch selection.

Bn and Bs are a lethal RNase and its inhibitor, respectively. The pink region indicates the region where cells die. Light blue indicates the area where the cells survive. On the left, by using a single switch, the overlap between non-target (red) and target cells (green) will be observed due to the leaky expression of Bn in the target cell. In contrast, by using dual switches (right), Bn and Bs expression levels from the co-transfected mRNAs show a proportional relationship on a two-dimensional plot. When the miRNA-ON switch encoding *Bn* and the miRNA-OFF switch encoding *Bs* are introduced, cells with high miRNA activity show the expression pattern indicated by the red dots. On the other hand, cells with weak or no miRNA activity show the expression pattern indicated by the green dots. When the total amount of transfected RNA changes, the expression pattern moves in the direction indicated by the double-edged arrows.

Supplementary Figure 6

A

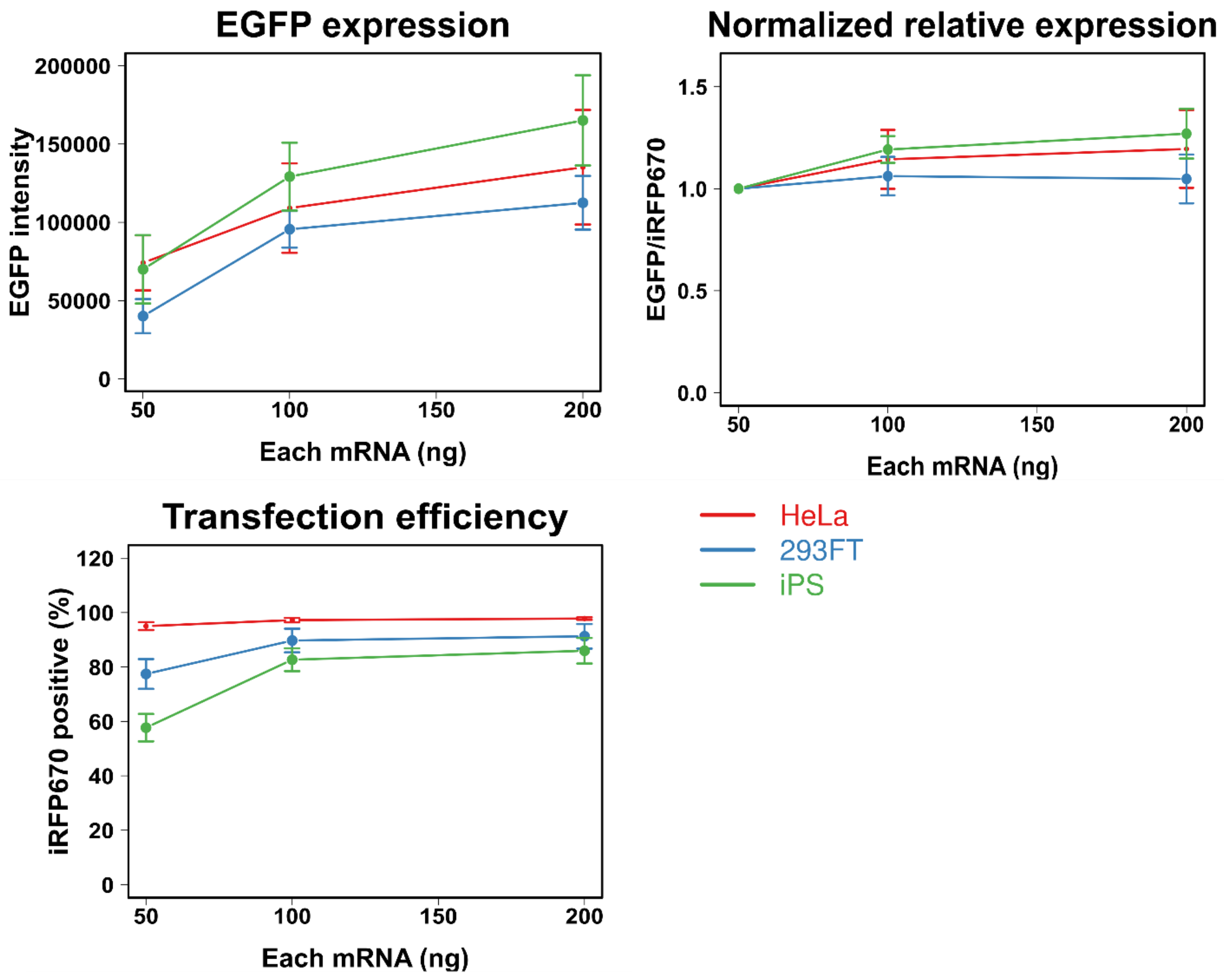

B

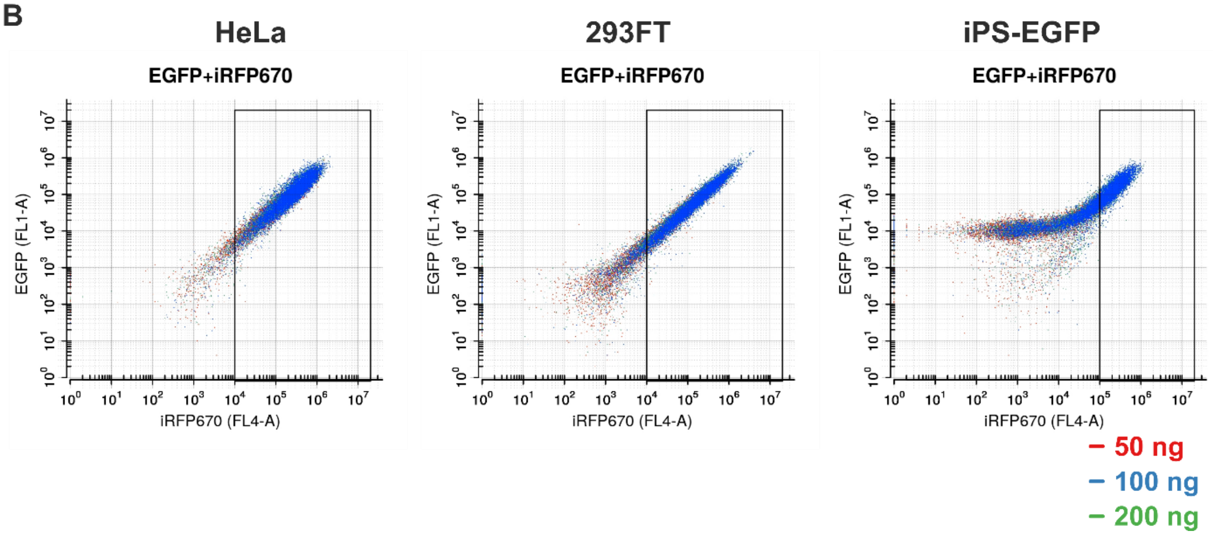

Fig. S6. Co-transfection ratio and efficiency of mRNAs

(A) The dose-dependency of the EGFP expression, relative expression (EGFP/iRFP670), and transfection efficiency in HeLa, 293FT and iPS cells. The cells were transfected with 50, 100, and 200 ng of *EGFP* mRNA and *iRFP670* mRNA (total 100, 200, and 400 ng, respectively). The relative expressions were normalized by the value of the cells transfected with 50 ng of each mRNA. **(B)** Scatter plots of cells co-transfected with *EGFP* mRNA and *iRFP670* mRNA. The cells inside the rectangles were defined as transfected cells and calculated for the expression level of EGFP. The iPS-EGFP cells originally showed a fluorescence intensity of 10^4. Changes in the fluorescence cannot be detected when the amount of transfected mRNAs is low (region where the co-transfected iRFP670 signal is less than 10^4), because the expression level of EGFP is too low compared with the originally expressed EGFP.

Supplementary Figure 7

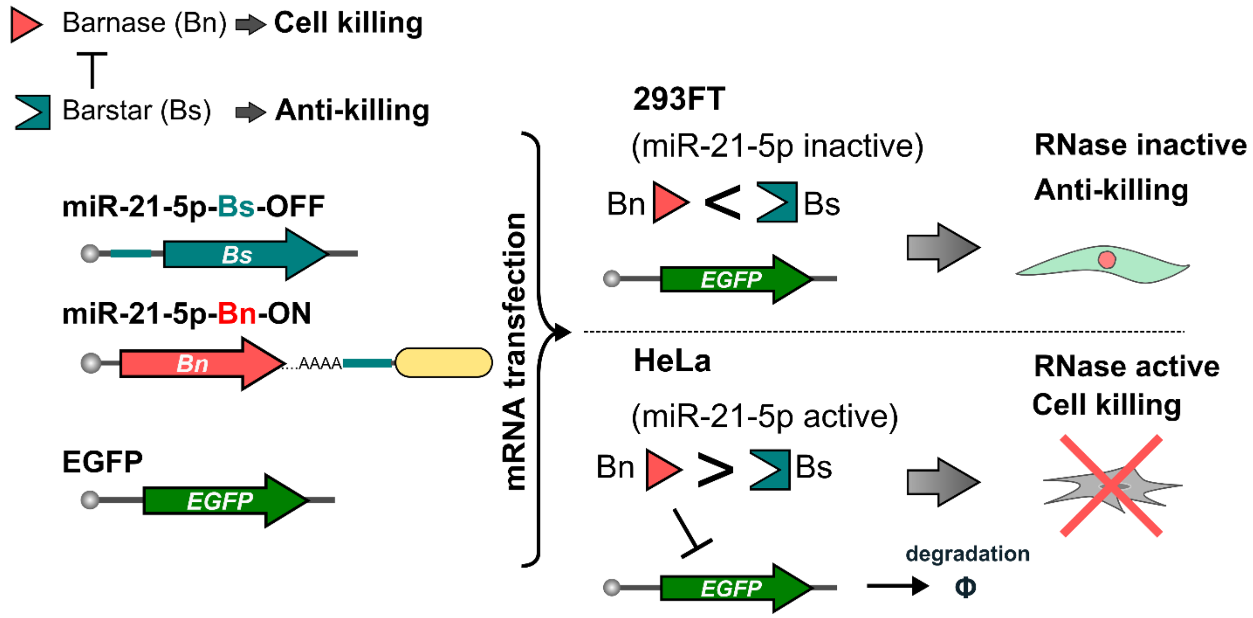

Fig. S7. Schematic illustration for detecting intracellular Bn activity via EGFP translation and selecting specific cell types.

The expression from miR-21-5p-OFF switch encoding *Bs* (miR-21-5p-Bs-OFF) and miR-21-5p-ON switch encoding *Bn* (miR-21-5p-Bn-ON) selectively kill HeLa cells because of the high miR-21-5p activity and high Bn activity. The active Bn in HeLa cells suppresses the EGFP translation from co-transfected *EGFP* mRNA. In contrast, the switches should not kill 293FT cells due to the high Bs activity.

Supplementary Figure 8

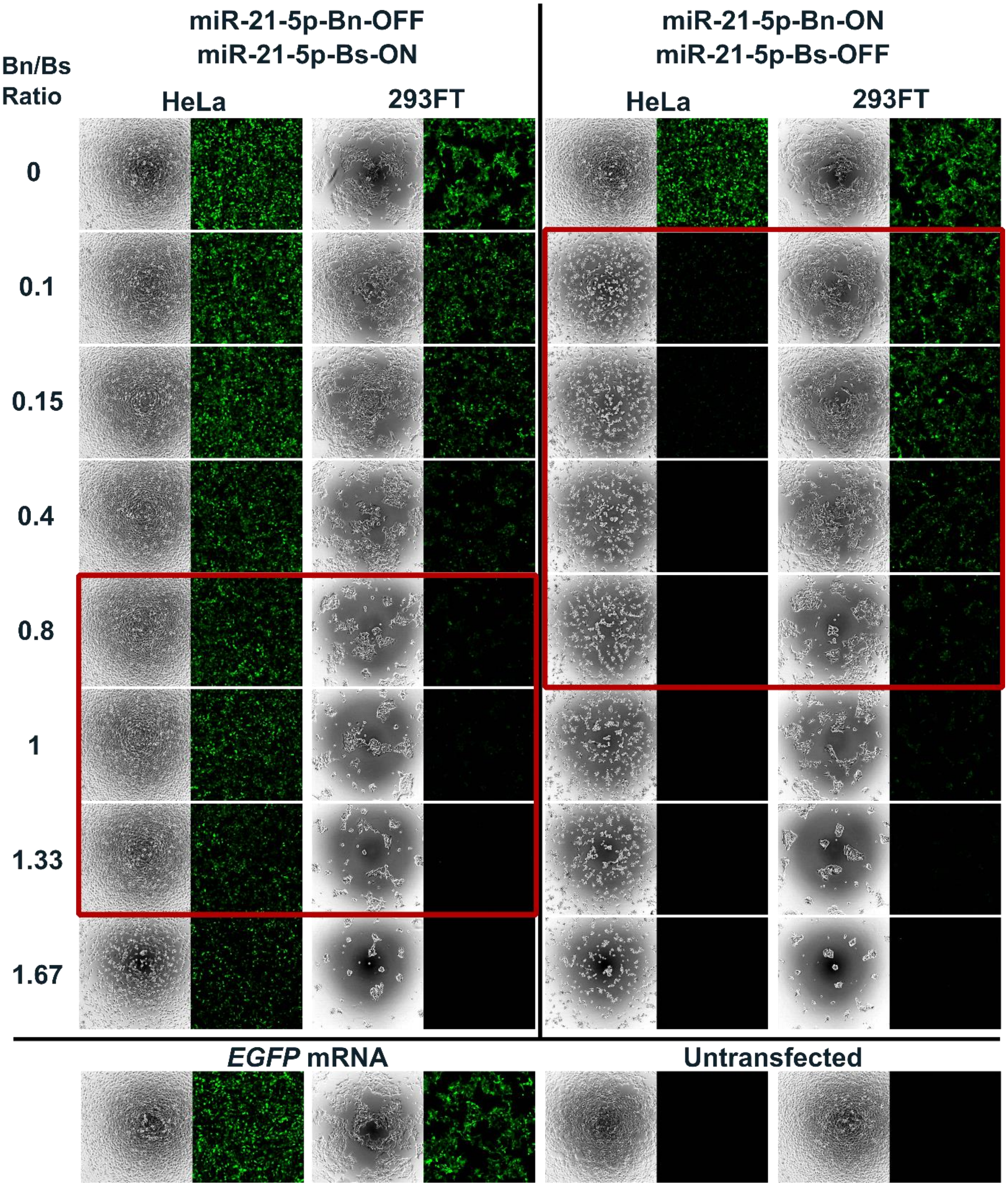

Fig. S8. Typical microscopic images to determine the criteria of the ratio and amount of the RNA switches.

HeLa and 293FT cells were transfected with miR-21-5p-Bn-OFF and -Bs-ON switches (or miR-21-5p-Bn-ON and -Bs-OFF switches) in addition to *EGFP* mRNA. The Bn/Bs ratio indicates the ratio of miR-21-5p-Bn and -Bs switches. The cells inside the red rectangles showed different EGFP expressions and cytotoxicity between HeLa and 293FT cells.

Supplementary Figure 9

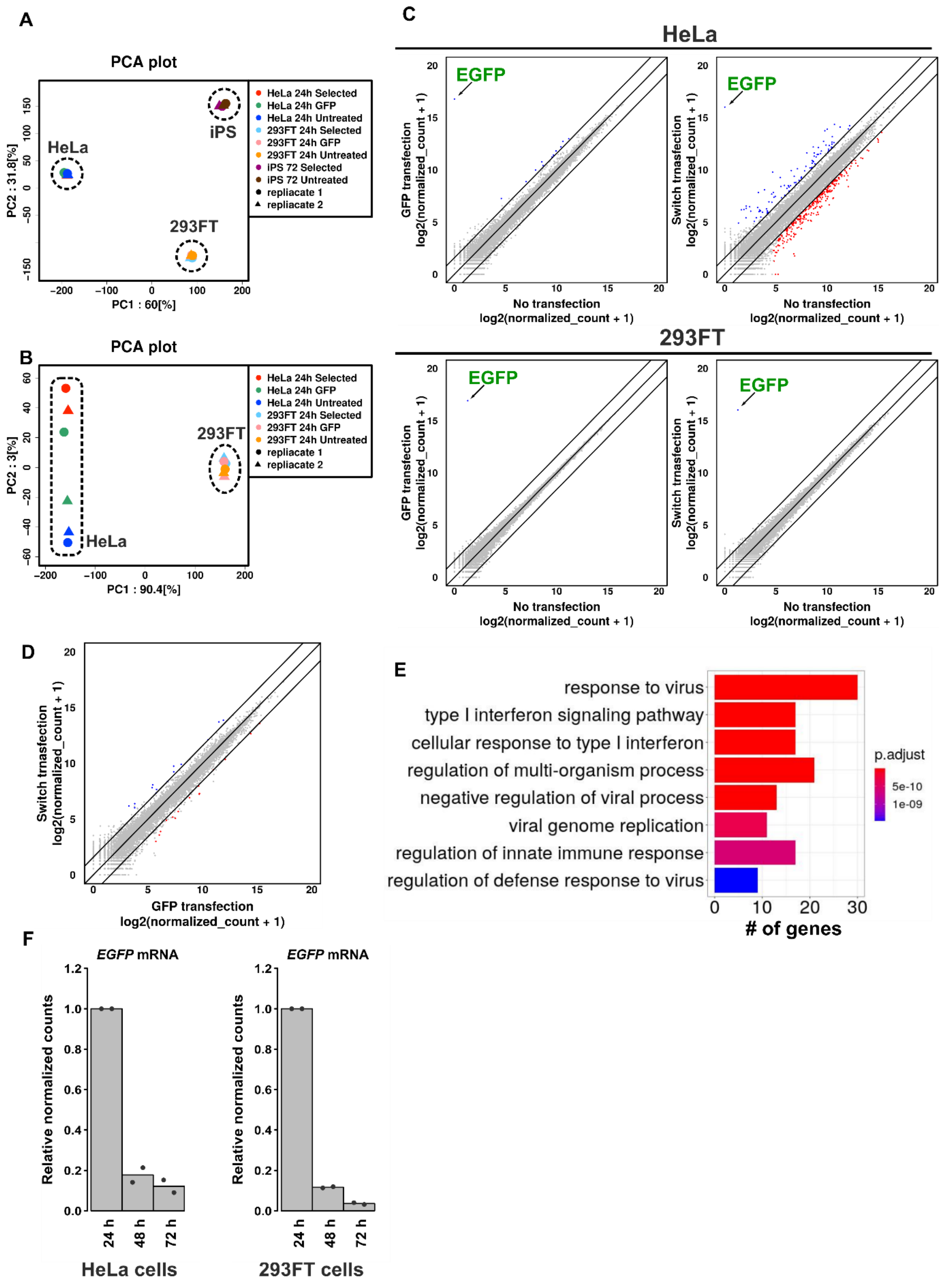

Fig. S9. Gene expression analysis of HeLa and 293FT cells transfected with EGFP mRNA or RNA switches.

(A) PCA biplots of HeLa, 293FT, and iPS cells. **(B)** PCA biplots of HeLa and 293FT cells to visualize the variation between HeLa samples. **(C)** Scatter plots of (normalized read counts) of HeLa and 293FT cells. Genes differentially expressed 3 times or more (padj < 0.05) compared with untransfected samples are shown in blue (higher) and red (lower). **(D)** Differentially expressed genes between HeLa cells transfected with *EGFP* mRNA or the switches. Genes differentially expressed 3 times or more (padj < 0.05) compared to cells transfected with *EGFP* mRNA are shown in blue (higher) and red (lower). **(E)** A gene ontology analysis of up-regulated genes in purified HeLa cells compared to untreated cells. **(F)** Relative read counts of *EGFP* mRNA normalized by the value at 24 hours after transfection. The data of each biological replicate (n=2) is shown as a point.

Supplementary Figure 10

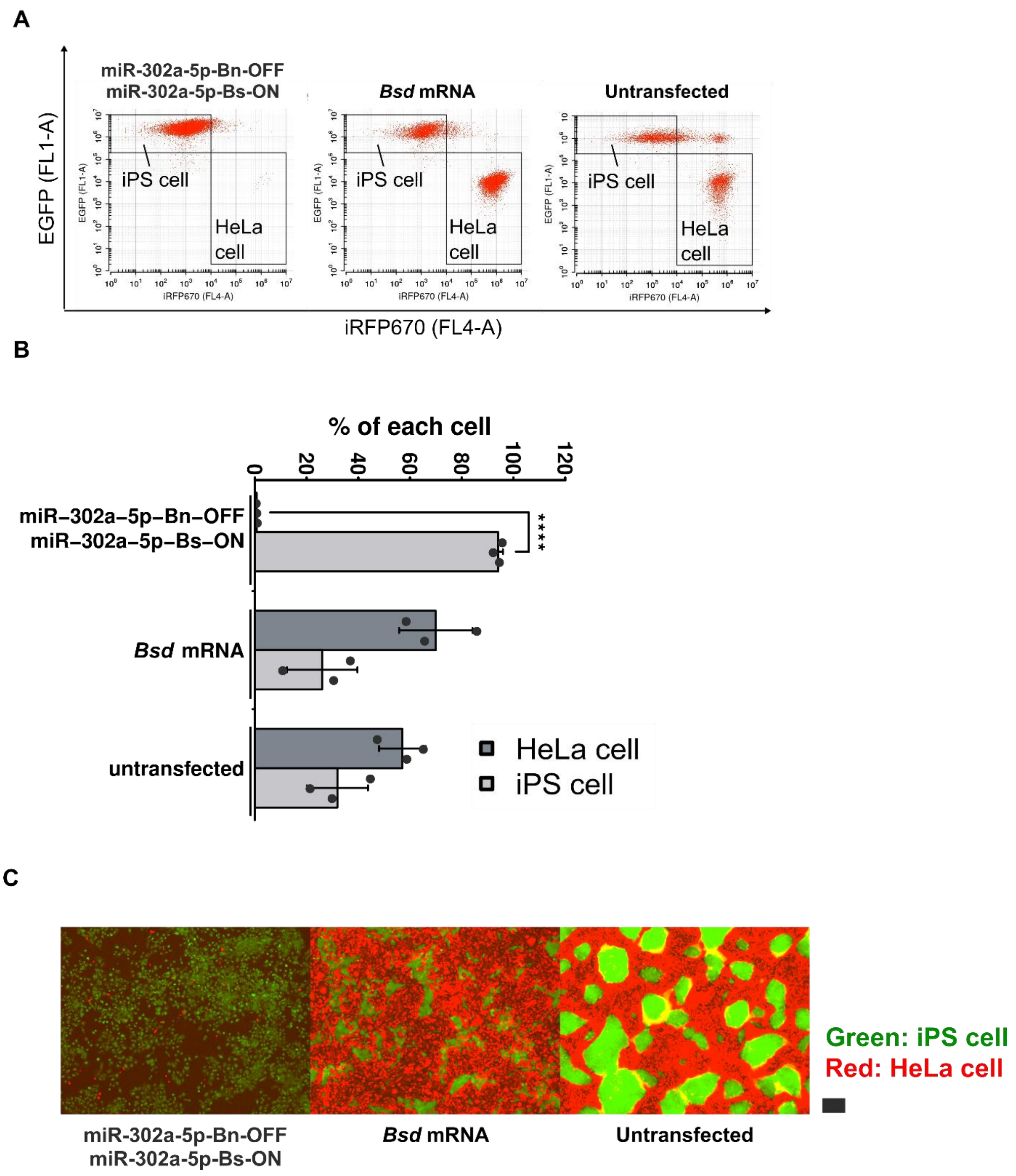

Fig. S10. Purification of iPSCs from a cell mixture without passage.

(A) Representative two-dimensional flow cytometry plots without passage. Co-cultured iPSCs expressing GFP and HeLa cells expressing iRFP670-M9 were treated with miR-302a-5p-OFF and -ON switches encoding *Barnase* and *Barstar*, respectively. At 3 days after the transfection,

all cells were analyzed by flow cytometry. The squares indicate the gates of iPSCs and HeLa cells, respectively. **(B)** Percentage of iPSCs and HeLa cells after transfection without passage. The number of cells for each cell type was counted inside the squares in (A), and the percentage of each cell line was calculated. Error bars represent the mean ± SD (n=3), and data of each biological replicate is shown as a point. ****P < 0.001. **(C)** Merged fluorescence images of the cells treated with the switches without passage. iPS-EGFP and HeLa-iRFP670-M9 are colored in green and red, respectively. Scale bar, 200 μm.

Supplementary Figure 11

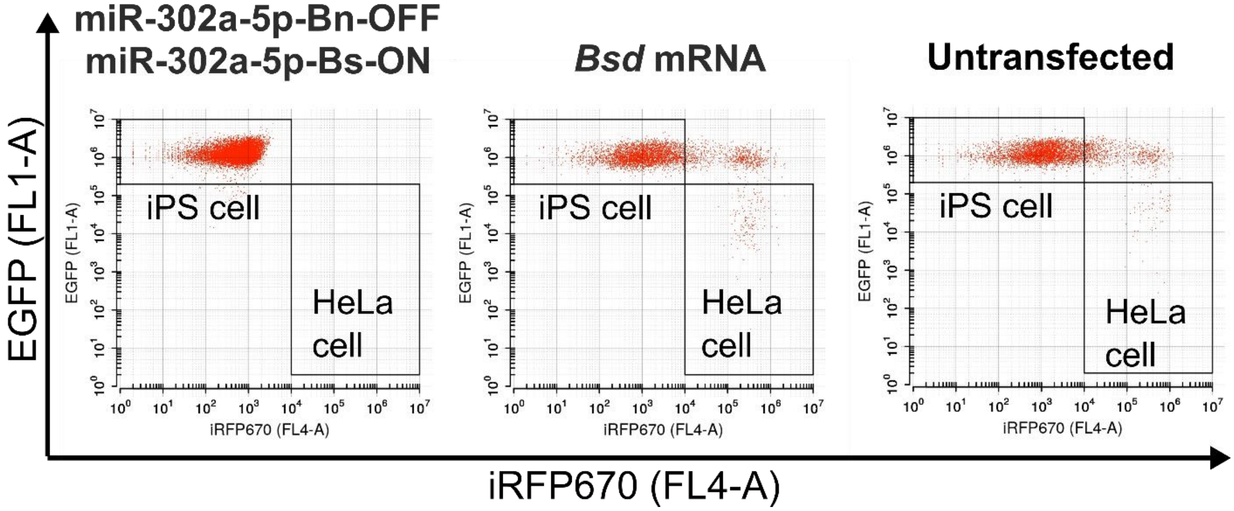

Fig. S11. Scatter plots of cells after purification by miR-302a-5p-ON and -OFF switches. Representative two-dimensional flow cytometry plots. Co-cultured iPSCs expressing GFP and HeLa cells expressing iRFP670-M9 were treated with miR-302a-5p-Bn-OFF and -Bs-ON switches. After the passage of the co-cultured cells, all cells were analyzed by flow cytometry. The squares indicate the gates of iPSCs and HeLa cells.

Supplementary Figure 12

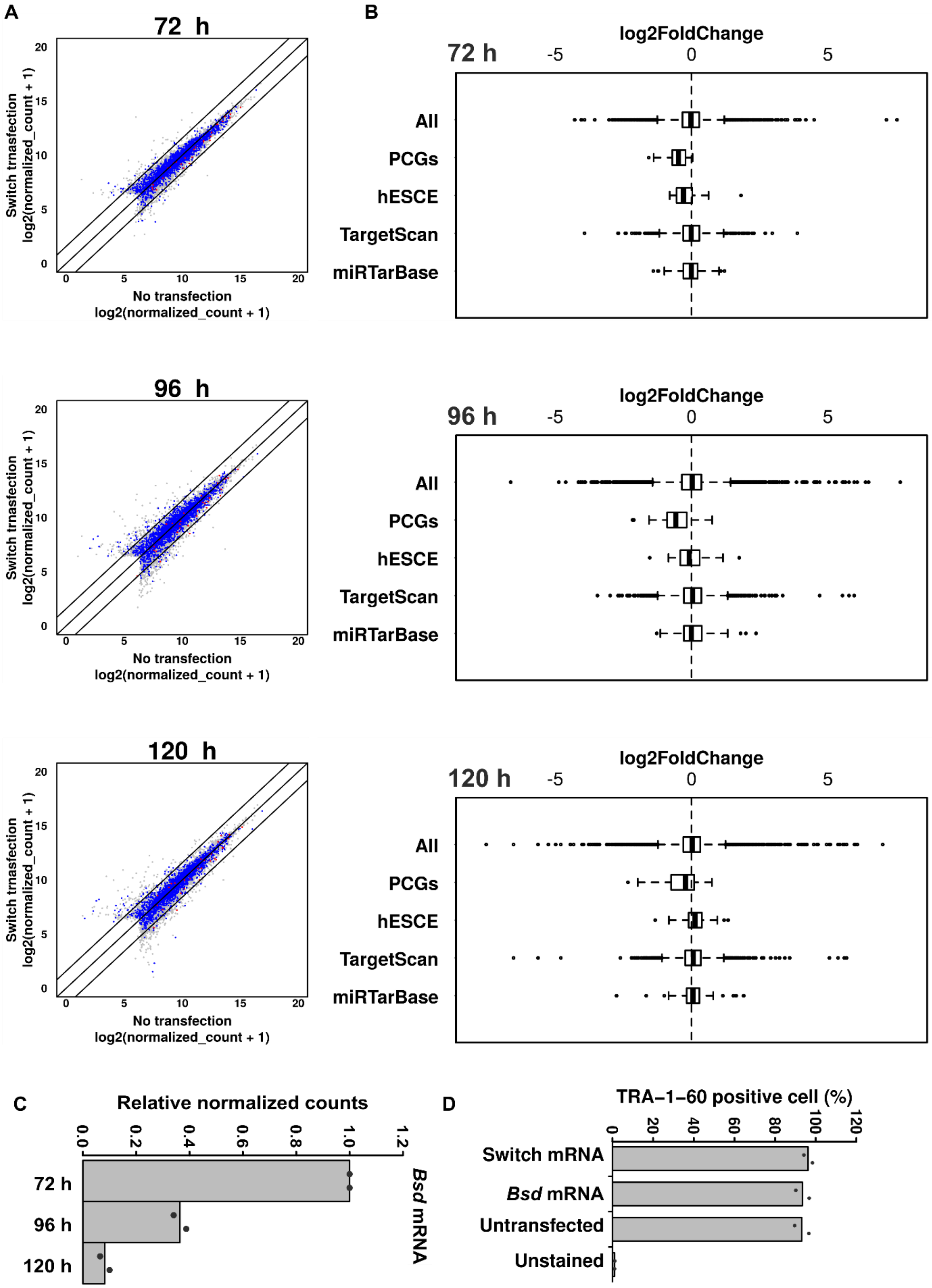

Fig. S12. Gene expression analysis of iPSCs transfected with RNA switches.

(A) Scatter plots of iPSCs at 72, 96 and 120 hours after selection. The miR-302a-5p-target genes predicted by TargetScan and pluripotency critical genes (PCGs) are highlighted in blue and red, respectively. The genes whose normalized expression level is less than 100 were excluded. **(B)** Boxplots of PCGs (*31*), human ESC essentialome genes (hESCE) (*32*), and target genes predicted by TargetScan (*29*) or miRTarBase (*30*). **(C)** Relative read counts of *Bsd* mRNA normalized by the value at 72 hours after the transfection. The data of each biological replicate (n=2) is shown as a point. **(D)** Mean percentage of TRA-1-60 positive cells at 10 days after the transfection with the three mRNAs (miR-302a-5p-OFF switch, miR-302a-5p-ON switch, and *Bsd* mRNA) (Switch mRNA). The cells transfected with mRNA encoding *Bsd* (*Bsd* mRNA) were used as transfection control. The data of each biological replicate (n=2) is shown as a point.

Supplementary Figure 13

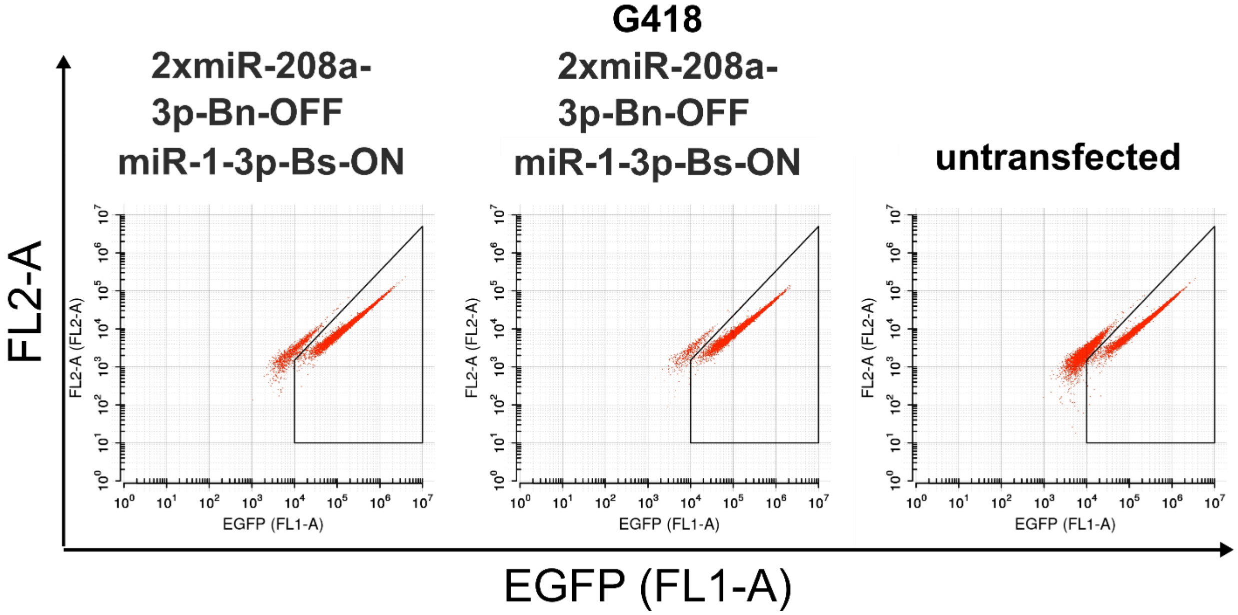

Fig. S13. Scatter plots of cells after purification by miR-208a-3p-Bn-OFF and miR-1-3p-Bs-ON.

Representative two-dimensional flow cytometry plots. The trapezoid indicates the gate of the cardiomyocytes expressing EGFP produced by the MYH6 promoter. 2 × miR-208a-3p-Bn-OFF contains 2 antisense sequences of the miR-208a-3p. G418 indicates cells treated with additional G418.

Supplementary Figure 14

A

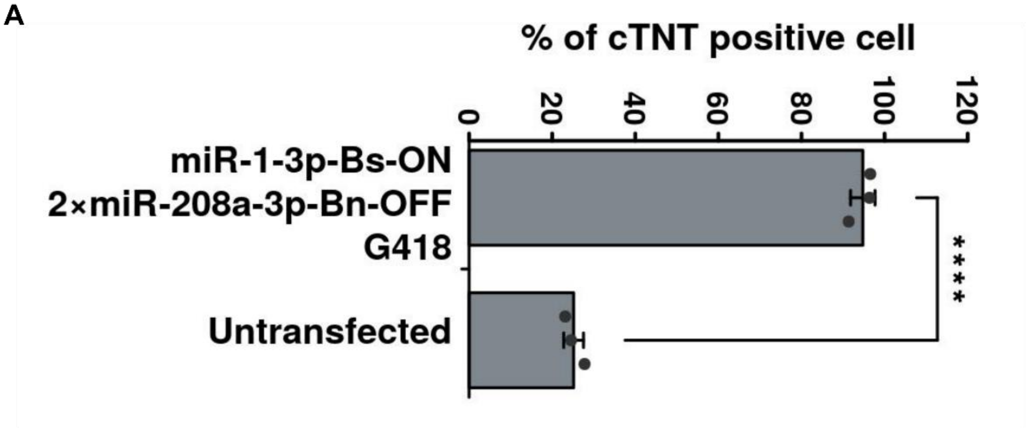

B

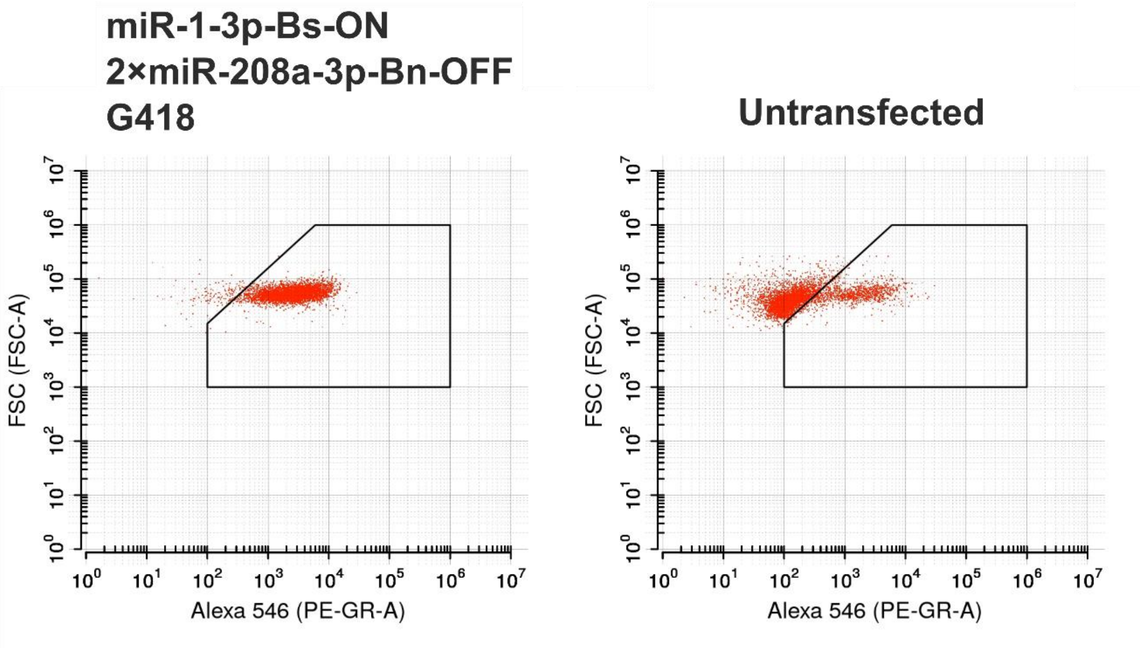

C

	Before purification	After purification
% of cardiomyocytes	63.33	94.8
Total cell counts	1,000,000	186,000
Cardiomyocytes counts	633,300	176,328
Yield (%)	100	27.8

Fig. S14. cTNT assay of purified iPSC-derived cardiomyocytes

(A) Percentage of iPSC-cardiomyocytes positive for cTNT after selection. Treatment with RNA switches (miR-208a-3p-Bn-OFF switch, and miR-1-3p-Bs-ON switch, *iRFP670* mRNA, and *aph* mRNA) increased the ratio of cTNT positive cells (94.8%) compared with untransfected cells (25.1%). Error bars represent the mean ± SD (n=3), and the data of each biological replicate (n=3) is shown as a point. ****P < 0.0005. **(B)** Representative scatter plots of cTNT stained cells after purification by miRNA-switches. The population in the area surrounded by the line is defined as cTNT positive cells. **(C)** Summary of the cells before and after the purification process.

Table S1. UTR sequences of the miRNA-ON switch.

The bold "aug" is the start codon. Underlined bold letters indicate antisense miR-21-5p or shuffled antisense miR-21-5p. The shuffled antisense miR-21-5p was used instead of antisense miR-21-5p for construction of the negative control ON switch. The poly(A) tail is shown as underlined letters.

5'UTR of ExCAG	GGGCAGCAGCAGCAGCAGCAGCAGCAGCAGCAGCAGCAGCAGCAGCAGCAGC<br>AGCAGCAGCAGCAGCAGCAGCAGCAGCAGCAGCAGCAGCAGAAUUAAGAGAG<br>AAAAGAAGAGUAAGAAGAAAUAUAAGACACCGGUcgccacc**aug**
3'UTR of ExCAG (CAG x 30)	uagucuagaccuucugcggggcuugccuucuggccaugcccuucuucucucccuugcaccuguaccucuu<br>ggucuuuGAAUAAAGCCugaguaggaaaaaaaaaaaaaaaaaaaaaaaaaaaaaaaaaaaaaaaaaaaa<br>aaaaaaaaaaaaaaaaaaaaaaaaaaaaaaaaaaaaaaaaaaaaaaaaaaaaaaaaaaaaaaaaaaaaaaaaaa<br>aau**ucaacaucagucugauaagcua**cauggucauagcuguuuccugCAGCAGCAGCAGCAGC<br>AGCAGCAGCAGCAGCAGCAGCAGCAGCAGCAGCAGCAGCAGCAGCAGCAGCA<br>GCAGCAGCAGCAGCAGCAGCAGGAAUUCUCGCAGAAA
5'UTR of Ex495nt and Ex1250nt	GGGCGAAUUAAGAGAGAAAAGAAGAGUAAGAAGAAAUAUAAGACACCGGUcgcc<br>acc**aug**
3'UTR of Ex495nt	uagucuagaccuucugcggggcuugccuucuggccaugcccuucuucucucccuugcaccuguaccucuu<br>ggucuuuGAAUAAAGCCugaguaggaaaaaaaaaaaaaaaaaaaaaaaaaaaaaaaaaaaaaaaaaaaa<br>aaaaaaaaaaaaaaaaaaaaaaaaaaaaaaaaaaaaaaaaaaaaaaaaaaaaaaaaaaaaaaaaaaaaaaaaaa<br>aau**ucaacaucagucugauaagcua**cauggucauagcuguUUCCUGUGUGAAAUUGUUAUC<br>CGCUCACAAUUCCACACAACAUACGAGCCGGAAGCAUAAAGUGUAAAGCCUG<br>GGGUGCCUAAUGAGUGAGCUAACUCACAUUAAUUGCGUUGCGCUCACUGCCC<br>GCUUUCCAGUCGGGAAACCUGUCGUGCCAGCUGCAUUAAUGAAUCGGCCAAC<br>GCGCGGGGAGAGGCGGUUUGCGUAUUGGGCGCUCUUCCGCUUCCUCGCUCA<br>CUGACUCGCUGCGCUCGGUCGUUCGGCUGCGGCGAGCGGUAUCAGCUCACU<br>CAAAGGCGGUCGCUUCCUCGCUCACUGACUCGCUGCGCUCGGUCGUUCGGC<br>UGCGGCGAGCGGUAUCAGCUCACUCAAAGGCGGUAAUACGGUUAUCCACAGA<br>AUCAGGGGAUAACGCAGGAAAGAACAUGUGAGCAAAAGGCCAGCAAAAGGCC<br>AGGAACCGUAAAAAGGCCGCGUUGCUGGCGUUUUUCCAUAGGCUCC
3'UTR of Ex1250nt	uagucuagaccuucugcggggcuugccuucuggccaugcccuucuucucucccuugcaccuguaccucuu<br>ggucuuuGAAUAAAGCCugaguaggaaaaaaaaaaaaaaaaaaaaaaaaaaaaaaaaaaaaaaaaaaaa<br>aaaaaaaaaaaaaaaaaaaaaaaaaaaaaaaaaaaaaaaaaaaaaaaaaaaaaaaaaaaaaaaaaaaaaaaaaa<br>aau**ucaacaucagucugauaagcua**cauggucauagcuguUUCCUGUGUGAAAUUGUUAUC<br>CGCUCACAAUUCCACACAACAUACGAGCCGGAAGCAUAAAGUGUAAAGCCUG<br>GGGUGCCUAAUGAGUGAGCUAACUCACAUUAAUUGCGUUGCGCUCACUGCCC<br>GCUUUCCAGUCGGGAAACCUGUCGUGCCAGCUGCAUUAAUGAAUCGGCCAAC<br>GCGCGGGGAGAGGCGGUUUGCGUAUUGGGCGCUCUUCCGCUUCCUCGCUCA<br>CUGACUCGCUGCGCUCGGUCGUUCGGCUGCGGCGAGCGGUAUCAGCUCACU<br>CAAAGGCGGUCGCUUCCUCGCUCACUGACUCGCUGCGCUCGGUCGUUCGGC<br>UGCGGCGAGCGGUAUCAGCUCACUCAAAGGCGGUAAUACGGUUAUCCACAGA<br>AUCAGGGGAUAACGCAGGAAAGAACAUGUGAGCAAAAGGCCAGCAAAAGGCC<br>AGGAACCGUAAAAAGGCCGCGUUGCUGGCGUUUUUCCAUAGGCUCCGCCCCC<br>CUGACGAGCAUCACAAAAAUCGACGCUCAAGUCAGAGGUGGCGAAACCCGAC<br>AGGACUAUAAAGAUACCAGGCGUUUCCCCCUGGAAGCUCCCUCGUGCGCUCU<br>CCUGUUCCGACCCUGCCGCUUACCGGAUACCUGUCCGCCUUUCUCCCUUCG<br>GGAAGCGUGGCGCUUUCUCAUAGCUCACGCUGUAGGUAUCUCAGUUCGGUG<br>UAGGUCGUUCGCUCCAAGCUGGGCUGUGUGCACGAACCCCCCGUUCAGCCC<br>GACCGCUGCGCCUUAUCCGGUAACUAUCGUCUUGAGUCCAACCCGGUAAGAC<br>ACGACUUAUCGCCACUGGCAGCAGCCACUGGUAACAGGAUUAGCAGAGCGAG<br>GUAUGUAGGCGGUGCUACAGAGUUCUUGAAGUGGUGGCCUAACUACGGCUA<br>CACUAGAAGAACAGUAUUUGGUAUCUGCGCUCUGCUGAAGCCAGUUACCUUC

	GGAAAAAGAGUUGGUAGCUCUUGAUCCGGCAAACAAACCACCGCUGGUAGCG<br>GUGGUUUUUUUGUUUGCAAGCAGCAGAUUACGCGCAGAAAAAAAGGAUCUCA<br>AGAAGAUCCUUUGAUCUUUUCUACGGGGUCUGACGCUCAGUGGAACGAAAAC<br>UCACGUUAAGGGAUUUUGGUCAUGAGAUUAUCAAAAAGGAUCUUCACCUAGA<br>UCCUUUUAAAUUAAAAAUGAAGUUUUAAAUCAAUCUAAAGUAUAUAUGAGUAA<br>ACUUGGUCUGACAGUUACCAAUGC
shuffled antisense of miR-21-5p	**<u>cuaauagguucauaaucgcaac</u>**

Table S2. Primer sequences

ID	name	sequence
KEC653	5UTRtemp_T302a-5p	CGACTCACTATAGGTTCCGCGATCGCGGATCCAGCAAGT<br>ACATCCACGTTTAAGTAGATCCACCGGTCGCCACCATG
KEC844	5UTRtemp_T21-5p	CGACTCACTATAGGTTCCGCGATCGCGGATCCTCAACAT<br>CAGTCTGATAAGCTAAGATCACACCGGTCGCCACCATG
KEC876	TAP_T7_1G	CAGTGAATTGTAATACGACTCACTATAG
KEC879	GCT7pro_5UTR2	GCTAATACGACTCACTATAGGTTCCTTAATCGCGGATCC
KEC883	3UTR120A	TTTTTTTTTTTTTTTTTTTTTTTTTTTTTTTTTTTTTTTTTTTT<br>TTTTTTTTTTTTTTTTTTTTTTTTTTTTTTTTTTTTTTTTTTTT<br>TTTTTTTTTTTTTTTTTTTTTTTTTTTTTTTTCCTACTCAGGC<br>TTTATTCA
YF472	Bs-1-Fwd	GACACCATGGGATCCAAAAAAGCAGTCATTAACGGGGAA<br>CAAATCAGAAGTATCAGC
YF477	Bs-6-Rev	TATGAGATCTGGAAAGTATGATGGTGATGTCGCAGCCTT<br>CCGCTTTCGCTTCAC
YF519	3UTR-109A-miR21	TAGCTTATCAGACTGATGTTGAATTTTTTTTTTTTTTTTTTT<br>TTTTTTTTTTTTTTTTTTTTTTTTTTTTTTTTTTTTTTTTTTTT<br>TTTTTTTTTTTTTTTTTTTTTTTTTTTTTTTTTTTTTTTTTTTT<br>TTCCTACTCAGGCTTTATTC
YF521	3UTR-109A-N	GTTGCGATTATGAACCTATTAGATTTTTTTTTTTTTTTTTTT<br>TTTTTTTTTTTTTTTTTTTTTTTTTTTTTTTTTTTTTTTTTTTT<br>TTTTTTTTTTTTTTTTTTTTTTTTTTTTTTTTTTTTTTTTTTTT<br>TTCCTACTCAGGCTTTATTC
YF565	M13Rev-Fwd	CATGGTCATAGCTGTTTCCTGTGTG
YF566	M13Rev-500-Rev	GGAGCCTATGGAAAAACGCCAGCAACG
YF567	M13Rev-1000-Rev	GCATTGGTAACTGTCAGACCAAGTTTACTC
YF578	Tg21-M13Rev	CACACAGGAAACAGCTATGACCATGTAGCTTATCAGACT<br>GATGTTGAATTT
YF579	N-M13Rev	CACACAGGAAACAGCTATGACCATGGTTGCGATTATGAA<br>CCTATTAGATTT
YF770	5UTR_fwd	GCGAATTAAGAGAGAAAAGAAGAGTAAGAAGAAATATAA<br>GACACCGGTCGCCACCATG
YF771	T7_5UTR_fwd	ATTGTAATACGACTCACTATAGGGCGAATTAAGAGAGAAA<br>AGAAGAGTAAG
YF772	Tg21-CAGx30	TTTCTGCGAGAATTCCTGCTGCTGCTGCTGCTGCTGCTG<br>CTGCTGCTGCTGCTGCTGCTGCTGCTGCTGCTGCTGCTG

		CTGCTGCTGCTGCTGCTGCTGCTGCTGCAGGAAACAGCTATGACCATGTAGCTTATCAGACTGATGTTGAAT
YF773	T7_CAGx30	ATTGTAATACGACTCACTATAGGGCAGCAGCAGCAGCAGCAGCAGCAGCAGCAGCAGCAGCAGCAGCAGCAGCAGCAGCAGCAGCAGCAGCAGCAGCAGCAGCAGCAGCAGCAGAATTAAGAGAGAAAAGAAGAGTAAG
YF783	Bs-ORF-Rev	GCCCCGCAGAAGGTCTAGACTAGGAAAGTATGATGGTGATG
YF804	Bs-ORF-Fwd	CACCGGTCGCCACCATGGGATCCAAAAAAGCAGTCATTAACGGG
YF805	Bs-pUCFus-Fwd	CTCTAGAGGATCCCCGTAAAAAAGCAGTCATTAACGG
YF806	Bs-Fus-Rev	TCTCTCTTAATTCGCGGAAAGTATGATGGTGATG
YF807	Rev3UTR2-pUCFus-Rev	TCGAGCTCGGTACCCCCTACTCAGGCTTTATTC
YF810	ORFComFwd	CACCGGTCGCCACCATG
YF811	ORFComRev	GCCCCGCAGAAGGTCTAGA
YF812	KW148-InvRev	TGGTGGCGACCGGTGGTACAAACTTGAATTAACCCTCAC
YF813	KW148-InvFwd	GACCTTCTGCGGGGCCACCCAGCTTTCTTGTACAAAG
YF818	5UTRtemp_2xT1-3p	CGACTCACTATAGGTTCCGCGATCGCGGATCCATACATACTTCTTTACATTCCAATACATACTTCTTTACATTCCAAGATCACACCGGTCGCCACCATG
YF819	5UTRtemp_2xT208a-3p	CGACTCACTATAGGTTCCGCGATCGCGGATCCACAAGCTTTTTGCTCGTCTTATACAAGCTTTTTGCTCGTCTTATAGATCACACCGGTCGCCACCATG
YF820	5UTRtemp_2xT9-5p	CGACTCACTATAGGTTCCGCGATCGCGGATCCTCATACAGCTAGATAACCAAAGATCATACAGCTAGATAACCAAAGAAGATCCACCGGTCGCCACCATG
YF821	5UTRtemp_2xT218-5pT2C	CGACTCACTATAGGTTCCGCGATCGCGGATCCACGTGGTTAGATCAAGCACAAACGTGGTTAGATCAAGCACAAAGATCAACACCGGTCGCCACCATG
YF822	3UTR-A109A-Tg1-3p	TGGAATGTAAAGAAGTATGTATTTTTTTTTTTTTTTTTTTTTTTTTTTTTTTTTTTTTTTTTTTTTTTTTTTTTTTTTTTTTTTTTTTTTTTTTTTTTTTTTTTTTTCCTACTCAGGCTTTATTC
YF823	3UTR-A109A-Tg208a-3p	ATAAGACGAGCAAAAAGCTTGTTTTTTTTTTTTTTTTTTTTTTTTTTTTTTTTTTTTTTTTTTTTTTTTTTTTTTTTTTTTTTTTTTTTTTTTTTTTTTTTTTTTTTTTCCTACTCAGGCTTTATTC

YF824	3UTR-A109A-Tg9-5p	TCTTTGGTTATCTAGCTGTATGATTTTTTTTTTTTTTTTTTT TTTTTTTTTTTTTTTTTTTTTTTTTTTTTTTTTTTTTTTTTTTT TTTTTTTTTTTTTTTTTTTTTTTTTTTTTTTTTTTTTTTTTTTT TTCCTACTCAGGCTTTATTC
YF825	3UTR-A109A-Tg218-5p	TTGTGCTTGATCTAACCATGTTTTTTTTTTTTTTTTTTTTTT TTTTTTTTTTTTTTTTTTTTTTTTTTTTTTTTTTTTTTTTTTTT TTTTTTTTTTTTTTTTTTTTTTTTTTTTTTTTTTTTTTTTTTTT TTCCTACTCAGGCTTTATTC
YF826	Tg1-3p-CAGx30	TTTCTGCGAGAATTCCTGCTGCTGCTGCTGCTGCTGCTG CTGCTGCTGCTGCTGCTGCTGCTGCTGCTGCTGCTGCTG CTGCTGCTGCTGCTGCTGCTGCTGCTGCAGGAAACAGCT ATGACCATGTGGAATGTAAAGAAGTATGTATTT
YF827	Tg208a-3p-CAGx30	TTTCTGCGAGAATTCCTGCTGCTGCTGCTGCTGCTGCTG CTGCTGCTGCTGCTGCTGCTGCTGCTGCTGCTGCTGCTG CTGCTGCTGCTGCTGCTGCTGCTGCTGCAGGAAACAGCT ATGACCATGATAAGACGAGCAAAAAGCTTGTTT
YF828	Tg9-5p-CAGx30	TTTCTGCGAGAATTCCTGCTGCTGCTGCTGCTGCTGCTG CTGCTGCTGCTGCTGCTGCTGCTGCTGCTGCTGCTGCTG CTGCTGCTGCTGCTGCTGCTGCTGCTGCAGGAAACAGCT ATGACCATGTCTTTGGTTATCTAGCTGTATGAT
YF829	Tg218-5p-CAGx30	TTTCTGCGAGAATTCCTGCTGCTGCTGCTGCTGCTGCTG CTGCTGCTGCTGCTGCTGCTGCTGCTGCTGCTGCTGCTG CTGCTGCTGCTGCTGCTGCTGCTGCTGCAGGAAACAGCT ATGACCATGTTGTGCTTGATCTAACCATGTTTT
YF958	Tg302a-5p-CAGx30	TTTCTGCGAGAATTCCTGCTGCTGCTGCTGCTGCTGCTG CTGCTGCTGCTGCTGCTGCTGCTGCTGCTGCTGCTGCTG CTGCTGCTGCTGCTGCTGCTGCTGCTGCAGGAAACAGCT ATGACCATGACTTAAACGTGGATGTACTTGCTT
YF959	3UTR-A109-Tg302a-5p	ACTTAAACGTGGATGTACTTGCTTTTTTTTTTTTTTTTTTTT TTTTTTTTTTTTTTTTTTTTTTTTTTTTTTTTTTTTTTTTTTTT TTTTTTTTTTTTTTTTTTTTTTTTTTTTTTTTTTTTTTTTTTTT TTCCTACTCAGGCTTTATTC

Table S3. List of primers and other DNA for IVT template and PCR fragment

Template for transcription or PCR fragment	primers 1	primer 2	primer 3	Other DNA
Normal mRNA template	YF771	KEC883		pUC19 vector including UTR and arbitrary ORF
miR-21-5p-OFF switch template	KEC879	KEC844	KEC883	pUC19 vector including UTR and arbitrary ORF
miR-1-3p-OFF switch template	KEC879	YF818	KEC883	pUC19 vector including UTR and arbitrary ORF
miR-9-5p-OFF switch template	KEC879	YF820	KEC883	pUC19 vector including UTR and arbitrary ORF
miR-208a-3p-OFF switch template	KEC879	YF819	KEC883	pUC19 vector including UTR and arbitrary ORF
miR-218-5p-OFF switch template	KEC879	YF821	KEC883	pUC19 vector including UTR and arbitrary ORF
miR-302a-5p-OFF switch template	KEC879	KEC653	KEC883	pUC19 vector including UTR and arbitrary ORF
495 bp fragment	YF565	YF566		pGEM-TAP
1250 bp fragment	YF565	YF567		pGEM-TAP
miR-21-5p-ON switch fragment 1	YF771	YF519	YF578	YFP0012-pUC19-*EGFP*
control ON switch fragment 1	YF771	YF521	YF579	YFP0012-pUC19-*EGFP*
miR-21-5p-ON switch (miR-21-495 nt) template	YF771	YF566		miR-21-5p-ON switch fragment 1<br>495 bp fragment
miR-21-5p-ON switch (miR-21-1250 nt) template	YF771	YF567		miR-21-5p-ON switch fragment 1<br>1250 bp fragment
control ON switch (shuffle-495 nt) template	YF771	YF566		control ON switch fragment 1<br>495 bp fragment
control ON switch (shuffle-1250 nt) template	YF771	YF567		control ON switch fragment 1<br>1250 bp fragment
CAGx30 EGFP ON switch fragment	YF773	YF519		YFP0012-pUC19-*EGFP*
miR-21-5p-ON switch (miR-21-ON-CAGx30) template	KEC876	YF772		CAGx30 EGFP ON switch fragment
miR-1-3p-ON switch fragment 1	YF773	YF822		YFP0012-pUC19-*EGFP*

miR-9-5p-ON switch fragment 1	YF773	YF824		YFP0012-pUC19-*EGFP*
miR-208a-3p-ON switch fragment 1	YF773	YF823		YFP0012-pUC19-*EGFP*
miR-218-5p-ON switch fragment 1	YF773	YF825		YFP0012-pUC19-*EGFP*
miR-302a-5p-ON switch fragment 1	YF773	YF959		YFP0012-pUC19-*EGFP*
miR-1-3p-ON switch template	YF773	YF826		miR-1-3p-ON switch fragment 1
miR-9-5p-ON switch template	YF773	YF828		miR-9-5p-ON switch fragment 1
miR-208a-3p-ON switch template	YF773	YF827		miR-208a-3p-ON switch fragment 1
miR-218-5p-ON switch template	YF773	YF829		miR-218-5p-ON switch fragment 1
miR-302a-5p-ON switch template	YF773	YF958		miR-302a-5p-ON switch fragment 1
Barstar ORF fragment 1	YF472	YF477		YF474, YF745, YF473, YF476
Barstar ORF for cloning	YF783	YF804		pCM-Bs
Barstar ORF	YF805	YF806		PCR fragment originated from Barstar ORF fragment 1
Barnase ORF	YF770	YF807		PCR fragment originated from pFN19K-HaloTag-T7SP6
miR-21-5p-Bn-OFF switch template	KEC879	KEC844	KEC883	YFP0042-pUC19-*Bs*-UTR-*Bn*
miR-21-5p-Bs-OFF switch template	KEC879	KEC844	KEC883	YFP0041-pUC19-*Bs*
miR-21-5p-Bn-ON switch fragment 1	YF773	YF519		YFP0042-pUC19-*Bs*-UTR-*Bn*
miR-21-5p-Bs-ON switch fragment 1	YF773	YF519		YFP0041-pUC19-*Bs*
miR-21-5p-Bn-ON switch template	KEC876	YF772		miR-21-5p-Bn-ON switch fragment 1
miR-21-5p-Bs-ON switch template	KEC876	YF772		miR-21-5p-Bs-ON switch fragment 1
miR-302a-5p-Bs-OFF switch template	KEC879	KEC653	KEC883	YFP0042-pUC19-*Bs*-UTR-*Bn*

miR-302a-5p-Bs-ON switch fragment 1	YF773	YF959		YFP0041-pUC19-*Bs*
miR-302a-5p-Bs-ON switch template	KEC876	YF958		miR-302a-5p-Bs-ON switch fragment 1
miR-208a-3p-Bn-OFF switch template	KEC879	YF819	KEC883	YFP0065-pUC19-*Bs*-UTR-PEST-*Bn*
miR-1-3p-Bs-ON switch fragment 1	YF773	YF822		YFP0041-pUC19-*Bs*
miR-1-3p-Bs-ON switch template	KEC876	YF826		miR-1-3p-Bs-ON switch fragment 1
Linearized piggyBac vector	YF812	YF813		KW148_PB53-CAG-GFP-puro
M9 fused hmAG1 or iRFP670	YF810	YF811		pUC19-*hmAG1-M9* or pUC19-*iRFP670*

Table S4. List of experimental conditions

Item	Cell	RNA	
Fig. 2A, B	HeLa (10,000 cells)<br>293FT (10,000 cells)	*EGFP* (switch or normal mRNA, 20 ng)	
		iRFP670 (internal control, 20 ng)	
Fig. 3A, B, C, D, fig. S1	HeLa (10,000 cells)<br>293FT (10,000 cells)	*EGFP* (switch or normal mRNA, 10 ng)	
		iRFP670 (internal control, 10 ng)	
		miRNA mimic/inhibitor (0.4 pmol)	
Fig. 4A, B, C, D	201B7 (20,000 cells)	*EGFP* (switch or normal mRNA, 10 ng)	
	293FT (10,000 cells)	*iRFP670* (internal control, 20 ng)	
Fig. 5A, B	HeLa (10,000 cells)<br>293FT (10,000 cells)	miR-21-5p-Bn-ON switch : miR-21-5p-Bs-OFF switch = 3:4 for selection of 293FT cells	
		miR-21-5p-Bn-OFF switch : miR-21-5p-Bs-ON switch = 4:3 for selection of HeLa cells	
Fig. 6B, C	HeLa-hmAG1-M9 (10,000 cells)<br>293FT-iRFP670-M9 (10,000 cells)	miR-21-5p-Bn-ON switch (3 ng) and miR-21-5p-Bs-OFF switch (27 ng) for selection of 293FT cells	*Bsd* (Blasticidin resistance gene, 5 ng)
		miR-21-5p-Bn-OFF switch (14 ng) and miR-21-5p-Bs-OFF switch (16 ng) for selection of HeLa cells	
		miR-21-5p-Bn-ON switch (3 ng) for single switch selection of 293FT cells	
		miR-21-5p-Bn-OFF switch (14 ng) for single switch selection of HeLa cells	
Fig. 7A, B, C	HeLa-hmAG1-M9: 293FT-iRFP670-M9 = 2:1 (total 10,000 cells)	miR-21-5p-Bn-ON switch (2 ng) and miR-21-5p-Bs-OFF switch (28 ng) for selection of 293FT cells	*Bsd* (Blasticidin resistance gene, 5 ng)
		miR-21-5p-Bn-OFF switch (14 ng) and miR-21-5p-Bs-OFF switch (16 ng) for selection of HeLa cells	

Fig. 8, fig. S10, S11	iPS:HeLa = 3:2 (total 120,000 cells)	miR-302a-5p-Bn-OFF switch (10 ng) and miR-302a-5p-Bs-ON switch (150 ng) for selection of iPSCs	*Bsd* (Blasticidin resistance gene, 100 ng)
Fig. 9, fig. S13	Differentiated cardiomyocytes (250,000 cells)	miR-208a-3p-Bn-OFF switch (112.5 ng) and miR-1-3p-Bs-ON switch (67.5 ng) for selection of cardiomyocytes	*iRFP670* (internal control, 50 ng), *aph* (G418 registance gene, 80 ng)
Fig. S4	HeLa-hmAG1-M9 (10,000 cells)<br>293FT-iRFP670-M9 (10,000 cells)	miR-21-5p-OFF 1-20 ng	
Fig. S6	HeLa (50,000 cells)<br>293FT (100,000 cells)<br>iPS-EGFP (100,000 cells)	*EGFP* mRNA (50, 100, 200 ng)<br>*iRFP670* mRNA (50, 100, 200 ng)	
Fig. S8	HeLa (10,000 cells)<br>293FT (10,000 cells)	7 ng of mixture of miR-21-5p-Bn or -Bs switches. The ratios are shown in Figure.	
Fig. S9	HeLa (100,000 cells)	miR-21-5p-Bn-OFF switch (22.8 ng), miR-21-5p-Bs-ON switch (17.2 ng) and EGFP mRNA (60 ng) for selection. (Bn:Bs=4:3)	
		EGFP mRNA (100 ng) for GFP transfected cells.	
	293FT (100,000 cells)	miR-21-5p-Bn-ON switch (3.6 ng), miR-21-5p-Bs-OFF switch (36.4 ng), and *EGFP* mRNA (60 ng) for selection (Bn:Bs=1:10)	
		EGFP mRNA (100 ng) for GFP transfected cells.	
Fig. S12	iPS (total 72,000 cells)	miR-302a-5p-Bn-OFF switch (10 ng) and miR-302a-5p-Bs-ON switch (150 ng) for selection of iPSCs	*Bsd* (Blasticidin resistance gene, 100 ng)

Fig. S14	Differentiated cardiomyocytes (1,000,000 cells)	miR-208a-3p-Bn-OFF switch (450 ng) and miR-1-3p-Bs-ON switch (270 ng) for selection of cardiomyocytes	*iRFP670* (internal control, 200 ng), *aph* (G418 registance gene, 320 ng)

Table S5. The mean number of analyzed cells

Fig. 2A

Sample name	HeLa	293FT
EGFP mRNA	6553	5171
miR-21-Ex495nt	6018	5112
miR-21-Ex1250nt	7548	5152
Shuffle-Ex495nt	5946	5076
Shuffle-Ex1250nt	6953	5134
miR-21-ExCAG	7165	5120

Fig. 3B

	miR-21-5p-EGFP-ON	miR-21-5p-EGFP-OFF
mimic	5572	5663
inhibitor	5678	5615
water	5626	5595

Fig. 3D

	miR-1-3p-ON switch	miR-9-5p-ON switch	miR-208a-3p-ON switch	miR-218-5p-ON switch
miR1-3p mimic	5318	5332	5656	5741
miR9-5p mimic	5379	5325	5812	5695
miR208a-3p mimic	5386	5082	5774	5528
miR218-5p mimic	5371	4911	5845	5231

Fig. 4D

	293FT	201B7
miR-302-5p-ON swich	5049	4698
miR-302-5p-OFF switch	5029	4549

Fig. 5B

Switch combination	total mRNA (ng)	HeLa	293FT
miR-21-5p-Bs-OFF + miR-21-5p-Bn-ON	30	5471	5132
	20	5469	4769
	10	5438	4732
	7	5447	4756
	5	5457	4932
	3	5494	5218
miR-21-5p-Bs-ON + miR-21-5p Bn-OFF	30	5187	4424
	20	5190	4078
	10	5137	4356
	7	5122	4359

	5	5090	4682
	3	5161	5238

Fig. 7C

Switch combination	HeLa	293FT
miR-21-5p-Bn-OFF + miR-21-5p-Bs-ON	5209	5141
miR-21-5p-Bn-ON + miR-21-5p-Bs-OFF	4756	4855
Bsd mRNA	4751	4709

Fig. 8B

miR-302a-5p-Bn-OFF + miR-302a-5p-Bs-ON	18562
Bsd mRNA	6184

Fig. 9C

miR-1-3p-Bs-ON + miR-208a-3p-Bn-OFF	15044
miR-1-3p-Bs-ON + miR-208a-3p-Bn-OFF + G418	13889
untransfected	20904

fig. S1

HeLa + miR-21-5p-EGFP-OFF	5595
293FT + miR-21-5p-EGFP-OFF	5208

fig. S4B

miR-21-5p-Bim-OFF (ng)	
0	4801
20	5114
15	4976
10	4895
5	4902
2	4884
1	4819

fig. S6

	50 ng	100 ng	200 ng
HeLa	10022	9835	9831
293FT	10176	10234	10215

iPS	10374	10387	10365

fig. S10B

miR-302a-5p-Bn-OFF + miR-302a-5p-Bs-ON	17803
Bsd	17160
untransfected	16139

fig. S12D

Switch mRNA	9944
Bsd mRNA	9999
Untransfected	9990
Unstained	10002

fig. S14

miR-1-3p-Bs-ON + miR-208a-3p-Bn-OFF + G418	5530
untransfected	6290
